# SCAMP3 Regulates EGFR and Promotes Proliferation and Migration of Triple-Negative Breast Cancer Cells through the Modulation of AKT, ERK, and STAT3 Signaling Pathways

**DOI:** 10.3390/cancers14112807

**Published:** 2022-06-05

**Authors:** Ariana Acevedo-Díaz, Beatriz M. Morales-Cabán, Astrid Zayas-Santiago, Michelle M. Martínez-Montemayor, Ivette J. Suárez-Arroyo

**Affiliations:** 1 Department of Biology, University of Puerto Rico, Bayamón, PR 00959, USA; ariana.acevedo3@upr.edu; 2 Department of Biochemistry, School of Medicine, Universidad Central del Caribe, Bayamón, PR 00960, USA; 419bmorales@uccaribe.edu (B.M.M.-C.); michelle.martinez@uccaribe.edu (M.M.M.-M.); 3 Department of Pathology, School of Medicine, Universidad Central del Caribe, Bayamón, PR 00960, USA; astrid.zayas@uccaribe.edu

**Keywords:** SCAMP3, triple-negative breast cancer, EGFR, ERK, PDGF, STAT3, AKT

## Abstract

**Simple Summary:**

Triple-negative breast cancer (TNBC) cases constitute ~15% of breast cancer tumors. This subtype is characterized by the lack of hormone receptors, estrogen and progesterone, and HER2, and it is a more aggressive, metastatic, and recurrent subtype than others. The need to develop novel anti-TNBC drugs encourages scientists to direct studies to identify potential therapeutic molecular targets. Our study aimed to define the role of the secretory carrier membrane protein, SCAMP3, in TNBC, and its association with the epidermal growth factor receptor (EGFR), which is present in ~90% of tumors. We confirmed that SCAMP3 promotes cell proliferation and motility and is directly associated with EGFR redistribution and degradation. It also regulates the gene and protein expression of molecules from the EGFR and PDGFR pathways. Finally, SCAMP3 depletion delays the establishment of TNBC tumors. This study provides evidence for SCAMP3′s TNBC tumor-promoting role and its potential as a target therapy for this disease.

**Abstract:**

Triple-negative breast cancer (TNBC) is the most aggressive, metastatic, and lethal breast cancer subtype. To improve the survival of TNBC patients, it is essential to explore new signaling pathways for the further development of effective drugs. This study aims to investigate the role of the secretory carrier membrane protein 3 (SCAMP3) in TNBC and its association with the epidermal growth factor receptor (EGFR). Through an internalization assay, we demonstrated that SCAMP3 colocalizes and redistributes EGFR from the cytoplasm to the perinucleus. Furthermore, SCAMP3 knockout decreased proliferation, colony and tumorsphere formation, cell migration, and invasion of TNBC cells. Immunoblots and degradation assays showed that SCAMP3 regulates EGFR through its degradation. In addition, SCAMP3 modulates AKT, ERK, and STAT3 signaling pathways. TNBC xenograft models showed that SCAMP3 depletion delayed tumor cell proliferation at the beginning of tumor development and modulated the expression of genes from the *PDGF* pathway. Additionally, analysis of TCGA data revealed elevated SCAMP3 expression in breast cancer tumors. Finally, patients with TNBC with high expression of SCAMP3 showed decreased RFS and DMFS. Our findings indicate that SCAMP3 could contribute to TNBC development through the regulation of multiple pathways and has the potential to be a target for breast cancer therapy.

## 1. Introduction

Breast cancer is the second leading cause of cancer-related deaths among women in the United States [1]. Triple-negative breast cancer (TNBC) is the most aggressive subtype of breast cancer, characterized by tumors that are negative for receptors of estrogen, progesterone, and human epidermal growth factor receptor 2 (HER2). TNBC constitutes ~15% of all breast cancer cases and is naturally recurrent and highly metastatic [2,3]. Despite currently available therapy for TNBC patients, the average survival rate is around ten months [3]. However, due to the heterogeneous characteristics of TNBC, specific treatment strategies for this disease are scarce. In fact, TNBC is managed with the use of conventional therapeutics, often leading to systemic relapse. Common treatments for TNBC involve alkylating agents, topoisomerase blockers, and taxanes [4]. Several signaling pathways have played a significant role in TNBC tumor initiation in the last decade. However, there is still a need to identify potential molecular targets to develop effective therapeutic agents.

Secretory carrier membrane protein 3 (SCAMP3) belongs to a family of integral protein components of the eukaryotic cell surface recycling system [5,6]. SCAMP3 controls the trafficking of epidermal growth factor receptor (EGFR) [6,7]. EGFR expression is markedly higher in TNBC compared to other breast cancer subtypes. It is present in 64% of TNBC tumors and is associated with poor clinical outcomes [8]. SCAMP3 sorts EGFR into multivesicular endosomes, enhancing EGFR recycling to the cell surface by negatively regulating its degradation [7]. Recently, studies have emerged to show the tumor-related role of SCAMP3 in a variety of cancers [9,10,11,12,13,14,15]. Previously, we published SCAMP3 expression on TNBC inflammatory breast cancer (IBC) cells, IBC patient tumor tissues, invasive embolus structure, and lymphatic vessels. Moreover, we identified the expression of the SCAMP3 protein in invasive ductal carcinomas [10]. However, the molecular basis for the SCAMP3 mechanism of action to regulate TNBC remains unexplored.

The present study investigated the relevance of SCAMP3 and EGFR signaling for the progression of TNBC by first defining the direct correlation between SCAMP3 and EGFR in our models. In this paper, we have revealed that SCAMP3 plays a key role in receptor internalization, redistribution, and degradation. The proliferation, cell survival, migration, and invasion of TNBC cells decreased after the depletion of SCAMP3 expression. Furthermore, tumor cell proliferation was impaired at the beginning of tumor development. We showed that SCAMP3 regulates EGFR, AKT, ERK, and STAT3 signaling pathways and modulates the expression of genes associated with platelet-derived growth factor (PDGF). Finally, analysis of The Cancer Genome Atlas (TCGA) data for gene expression revealed that high SCAMP3 expression in patients with TNBC is associated with low relapse-free survival (RFS) and distant metastasis-free survival (DMFS). For the first time, these findings demonstrate the tumor-promoting role of SCAMP3 in TNBC and its association with EGFR and other receptor tyrosine kinases and expose the potential to develop SCAMP3-targeted anticancer therapies to increase patient survival.

## 2. Materials and Methods

### 2.1. Cell Culture and Reagents

MDA-MB-468 and MDA-MB-231 cell lines were obtained from American Type Culture Collection (ATCC^®^, Manassas, VA, USA) and cultured in Dulbecco’s Modified Eagle Medium (DMEM) (Gibco/Life Technologies, Waltham, MA, USA) supplemented with 10% fetal bovine serum (FBS) and penicillin-streptomycin-glutamine. SUM-149 cells were obtained from (BiolVT, Westbury, NY, USA) and cultured in Ham’s F-12 Nutrient Mixture (Gibco/Life Technologies, Waltham, MA, USA) supplemented with 10% FBS. MCF-10A cells (ATCC^®^, Manassas, VA, USA) were cultured in DMEM/Ham’s F-12 with 10% horse serum (HS) (Sigma Aldrich, St. Louis, MO, USA) and supplemented with 20 ng/mL of Epidermal Growth Factor (EGF), Cholera Toxin B, Hydrocortisone solution, HEPES and insulin-transferrin-sodium. All cell lines used in this study were authenticated by short tandem repeat (STR) profiling, and Mycoplasma detection was screened with the Mycoplasma Detection Kit (Nordic BioSite AB, Täby, Stockholm, Sweden) before use. The 5 mM working stock of erlotinib (MedChemExpress, Monmouth Junction, NJ, USA) was dissolved in 100% sterile DMSO (Sigma Aldrich, St. Louis, MO, USA).

### 2.2. Plasmids, Lentiviral Transduction, and Transfections

Lentiviral transduction: HEK-293T were grown to 60–70% confluence in a 60 mm plate, then transfected with sgRNA (CRISPR/Cas9 system lentiCRISPRv2.0 backbone, plasmid 52961) (Addgene, Watertown, MA, USA) or lentiviral expression vectors (LV-h-SCAMP3 ORF) (Vector Builder, Chicago, IL, USA) together with packaging plasmids (PCMV delta R8.2 and pMD2. G (Adggene, Watertown, MA, USA). FuGENE HD (Promega) was used as the transfection reagent according to the manufacturer’s instructions. After 18 h posttransfection, cells were refreshed with DMEM supplemented with 30% FBS for viral particle stability and the supernatant was collected at 48 h. The cells were refreshed again, and the supernatant was collected the next day. Finally, the particles were concentrated using the Lenti-X™ Concentrator (Takara, Kusatsu, Shiga, Japan) for 72 h and stored at −80 °C. Cancer cells were transduced with the virus and selected in puromycin.

siRNA transfections: MDA-MB-231 cells were seeded in a six-well plate until 70% confluent. Cells were transfected with SCAMP3 targeting siRNA and nontargeting sequences (control) at a final concentration of 50 nM using FuGENE HD^®^ (Promega, Madison, WI, USA). SCAMP3 siRNA (sc-41294) and scrambled siRNA (sc-37007) were purchased from Santa Cruz Biotechnology (Dallas, TX, USA).

### 2.3. Proliferation Assay

Parental or SCAMP3 silenced breast cancer cells were seeded in a 96-well plate with a density of 2.0 × 10^3^ cells/well. MCF-10A controls or SCAMP3 overexpressing cells were seeded with a density of 1 × 10^5^ cells/well in a 24 well plate for 24, 48, and 72 h. Proliferation was assessed using the CyQUANT^®^ NF Cell Proliferation Assay Kit (Invitrogen, Waltham, MA, USA). Fluorescence was measured using a GloMax^®^ microplate reader (Promega, Madison, WI, USA) at 530 nm. The experiments were carried out in triplicate at least three times.

### 2.4. MTT Cell Proliferation Assay

Cells were starved for 24 h before the experiments. The next day, cells were stimulated with 10 ng/mL EGF for 30 min and seeded at 5 × 10^4^ cells in each well of 48-well plates in triplicate and incubated for 72 h at 37 °C and 5% CO_2_. After the incubation period, using the Cell Proliferation Kit I (MTT) (Roche, Basel, Switzerland) to measure proliferation in response to EGF, a final concentration of 0.5 mg/mL of the MTT labeling reagent was added to each well. The microplate was then incubated for 4 h. After incubation, 150 μL of the solubilization solution was added to each well and the plate was allowed to stand overnight in the incubator. The microplate reader was used to measure the absorbance of the formazan product at 600 nm. The reference wavelength was measured at 650 nm. Cell proliferation was calculated as the percentage of SCAMP3 knockout cells relative to wild type.

### 2.5. Migration and Invasion Assays

Cell capacity to migrate and invade was measured using Corning^®^ FluoroBlokTM cell culture inserts (BD Biosciences, San José, CA, USA) and the BD BioCoat Matrigel^TM^ invasion assay (BD Biosciences, San José, CA, USA), respectively. Quiescent 1.5 × 10^5^ SUM-149 cells were seeded in the upper chambers, then stimulated with 10 ng/mL EGF and incubated at 37 °C to allow invasion/migration to 10% FBS medium (chemoattractant). After 24 h, cells on the upper membrane surface were removed with a cotton swab. Cells attached to the bottom surface of the membrane were fixed and stained with propidium iodide (Sigma Aldrich, St. Louis, MO, USA). Cells were quantified with v1.48 (NIH, Bethesda, MD, USA). Micrographs were obtained at a magnification of 200× with an Olympus inverted fluorescence microscope (Center Valley, PA, USA).

### 2.6. Wound Healing

1.2 × 10^5^ MCF-10A control cells and SCAMP3 overexpressing cells were cultured in complete medium (10% HS) in two-well silicone inserts with a defined cell-free gap wound plate (Ibidi ^®^ Inc., Fitchburg, WI, USA) for 24 h. Then the medium was changed to the experimental medium (no EGF, 2% HS). Twenty-four hours after incubation, the insert was removed, and cells were allowed to migrate for 24 h. The cells were then fixed with 4% paraformaldehyde for 15 min and washed with 1× PBS. Subsequently, cells were permeabilized with 0.5% Triton X-100 for 10 min at room temperature and blocked with 1% BSA. Rhodamine phalloidin (Life Technologies, Waltham, MA, USA) was used to stain F-actin. After three washes with 1× PBS, cells were incubated for 1 min at room temperature with 1 µg/mL of DAPI (Life Technologies) to stain the nuclei. The width of the wound was determined by measuring the distance (μm) between the edges of the wound using cellSens Imaging software (Olympus Corp., Center Valley, PA, USA).

### 2.7. EGFR Internalization Assay

To observe EGF internalization, 1.2 × 10^5^ SUM-149, MDA-MB-468 WT, and their respective knockout cells were seeded on coverslips and placed in starvation for 24 h. Then, 10 ng/mL of EGF was added at 0, 15, 30, and 60 min. After each time point, the cell plates were placed on ice and washed with 1× PBS. After incubation, cells were fixed with 4% paraformaldehyde for 15 min, washed with 1× PBS, and permeabilized with 0.5% Triton X-100 for 10 min. The cells were then blocked with 5% BSA for 1 h at room temperature. For immunolabeling, fixed and permeabilized cells were incubated for 2 h with primary antibodies (EGFR monoclonal antibody (199.12) (1:250, #MA5-13319) and polyclonal SCAMP3 antibody (1:250, #PA5-21428) (ThermoFisher, Waltham, MA, USA). After washing, coverslips were incubated with anti-rabbit Alexa 488 (1:750, #4412, Cell Signaling Technology (CST), Danvers, MA, USA) and anti-mouse Alexa 594 (1:750, #8527, CST) for 1 h at room temperature. After washes, cells were incubated for 1 min at room temperature with 1 µg/mL of DAPI (ThermoFisher) and washed. Cells were mounted on slides with an antifade medium (ThermoFisher). Micrographs were acquired by confocal microscopy with an Olympus BX60 microscope (Olympus; Tokyo, Japan). Cell total fluorescence (CTF) was quantified as follows: CTF = integrated density − (Area of selected cell × mean fluorescence of background readings). The percentage of colocalization was calculated by dividing the area of colocalization between the area of total cells multiplied by 100.

### 2.8. Colony Formation Assay

Wild-type or SCAMP3 silenced breast cancer cells were seeded in a 24-well plate with a seeding density of 200 (SUM-149) or 400 cells/well (MDA-MB-468), respectively. Ten days later, colonies were fixed with methanol, washed with 1× PBS, and stained with crystal violet (Sigma Aldrich, St. Louis, MO, USA). Colonies with ≥50 cells were counted manually and analyzed.

### 2.9. Immunoblotting

Cells were lysed in lysis buffer (10% SDS, 10% sodium deoxycholate, 1% Triton-X 100, 1% Igepal, and cOmplete^TM^ Mini Protease Inhibitor Cocktail (Sigma Aldrich, St. Louis, MO, USA). Total protein was quantified using the Precision Red reagent (Cytoskeleton, Denver, CO, USA), subjected to separation by SDS-PAGE gels, and transferred onto a PVDF membrane. After blocking with 5% milk, the membranes were incubated with the indicated primary antibodies against: AKT (#9272, CST, Danvers, MA, USA), p-AKT (Ser473) (#4060, CST), p44/42 MAPK (ERK1/2) (#9102, CST), phospho-p44/42 MAPK (p-Erk1/2 Thr202/Tyr204) (#4370, CST), EGFR (#4267S, CST), pSTAT3 (Tyr705) (#9145, CST), STAT3 (#4904, CST) and anti- β-tubulin (#86298, CST) and SCAMP3 (#PA5-21428) (Invitrogen, Waltham, MA, USA). Anti-pEGFR (Tyr1068) was acquired from Santa Cruz Biotechnology and anti -β-Actin from Sigma Aldrich (St. Louis, MO, USA). The membranes were then incubated with secondary antibodies for 1 h. Immunoblot detection and quantitation were carried out with Pierce™ ECL Western Blotting Substrate kit (ThermoFisher, Waltham, MA, USA) and BioSpectrum Imaging System (UVP LLC, Upland, CA, USA).

### 2.10. Tumorsphere Formation Assay

SUM-149 cells were seeded at a density of 1 × 10^4^ cells/mL with 2% polyethylene glycol (Sigma Aldrich, St. Louis, MO, USA) in 6-well ultralow attachment plates (Corning^®^, Corning, NY, USA) and incubated for 72 h. Micrographs were captured using cellSens Imaging software (Olympus Corp., Center Valley, PA, USA). Tumorsphere number and area were calculated using Image J v1.48 (NIH, Bethesda, MD, USA).

### 2.11. EGFR Degradation Assay

SUM-149 cells were cultured in a 6 well plate and incubated until they reached 80% confluency. Subsequently, cells were starved for 24 h and then treated with 100 μM cycloheximide (Sigma Aldrich, St. Louis, MO, USA) for 1 h to inhibit protein synthesis. Cell lysates were prepared after stimulation with 10 ng/mL of EGF for 0, 0.5, 1, 2, and 4 h. Cells were lysed, total protein was quantified using Precision Red reagent (cytoskeleton), and the expression of EGFR and SCAMP3 protein was evaluated by immunoblotting.

### 2.12. RNA Isolation and Quantitative Real-Time RT-PCR (RT-qPCR) Assays

Gene expression profiles were obtained from wild-type SUM-149 and SCAMP3 knockout cells or 30 mg of tumor tissue extracted from mice injected with both cell lines. Total RNA extraction and gDNA elimination were performed using the Qiagen RNeasy Kit (Qiagen, Hilden, Germany). RNA concentration was detected using a NanoDrop (NanoDrop Technologies, Wilmington, DE, USA). Then, 500 ng of RNA was used to synthesize cDNA using the C-03 RT^2^ First Strand Kit (Qiagen, Hilden, Germany), and gene expression profiles of 84 genes were investigated using the human EGF/ PDGF Signaling Pathway (PAHS-040ZD-12) RT^2^ Profiler^TM^ PCR arrays (Qiagen, Hilden, Germany). Gene expression levels were individually evaluated using the 2^(−2ΔCt)^ formula by comparing the relative gene expression of 84 genes with reference genes (*ACTB*, *B2M*, *GAPDH*, *HPRT1*, *RPLP0*) [16]. Reproducibility was maintained using three biological replicates from three individual experiments or three different tumors.

### 2.13. In Vivo Study

The study was approved by the Institutional Animal Care and Use Committee of the UCC (IACUC) and was carried out following the IACUC guidelines. 21d female SCID mice (Charles River Laboratories International, Wilmington, MA, USA)] were housed under specific pathogen-free conditions and fed with 2920X Teklad Global Rodent Diet (Harlan Laboratories, Indianapolis, IN, USA), and sterile water was provided ad libitum. To test the effects of SCAMP3 on tumor formation and progression, we injected 3.0 × 10^6^ wild-type SUM-149 and SCAMP3 knockout cells in 1:1 serum free-medium: Matrigel on the mammary fat pad of mice. After tumor establishment, the group allocation was randomly made: (a) mice injected with wild-type cells (*n* = 9) and (b) mice injected with SCAMP3 silenced cells (*n* = 9). One week after injection, mouse weight and tumor volume were measured weekly for 10 weeks. Tumor volume (mm^3^) was measured with a caliper and calculated as follows: [(width)^2^ × length]/2. At the end of the study, the mice were sacrificed, and the tumors were excised and maintained in optimal conditions for future experiments.

### 2.14. Bioinformatic Analysis

SCAMP3 expression in breast cancer was evaluated in breast cancer patients included in the Cancer Genome Atlas (TCGA) database using the UALCAN database (http://ualcan.path.uab.edu) accessed on 22 May 2022 [17]. Survival data were evaluated in a total of 1075 breast cancer patients with low (*n* = 855) and high (*n* = 220) expression of SCAMP3 using The Human Protein Atlas database (https://www.proteinatlas.org/ENSG00000116521-SCAMP3/pathology/breast+cancer) accessed on 23 May 2022 [18]. Relapse-free survival (RFS) and distant metastasis-free survival (DMFS) data in TNBC were analyzed using the Kaplan–Meier Plotter database (https://kmplot.com) accessed on 23 May 2022 [19]. Survival data are derived from Gene Expression Omnibus (GEO), European Genome-phenome Atlas (EGA), and TCGA. The prognosis of each group of patients was examined using Kaplan–Meier survival estimators, and the survival outcomes of the two groups were compared using log-rank tests. The correlation of SCAMP3 with EGFR or STAT3 was identified using the cBioPortal web server (https://www.cbioportal.org/) in breast invasive carcinoma (TCGA, Firehouse Legacy) accessed on 23 May 2022.

### 2.15. Statistical Analysis

Data are expressed as mean ± S.E.M. for at least three independent experiments. In vitro statistical analyses were performed using Student’s *t*-test or two-way analysis of variance (ANOVA). Statistical analyses were done using Graph Pad Prism 9.0 (San Diego, CA, USA) and are considered significant when *p* ≤ 0.05. Gene expression studies in cells or tumors were individually evaluated using the 2^(−^^Δ^^Ct)^ formula by comparing their relative gene expression to the expression of reference genes. The *p* values for the gene expression PCR array analysis were calculated based on a Student’s *t*-test of the replicate 2^(−^^Δ^^Ct)^ values for each gene in the wild type group and the SC3KO group following the manufacturer’s instructions.

In vivo studies: Initially, the average differences in tumor volume according to the condition were evaluated, considering the effect of the week through a mixed linear regression model. Comparisons of the average tumor size by group and week were evaluated by unpaired t-test assuming unequal variances. StataCorp. 2019. Stata Statistical Software: Release 16. College Station, TX, USA: StataCorp LLC.

## 3. Results

### 3.1. Low SCAMP3 Expression Is Associated with Decreased Proliferation, Colony, and Tumorsphere Formation

**Figure 1:**
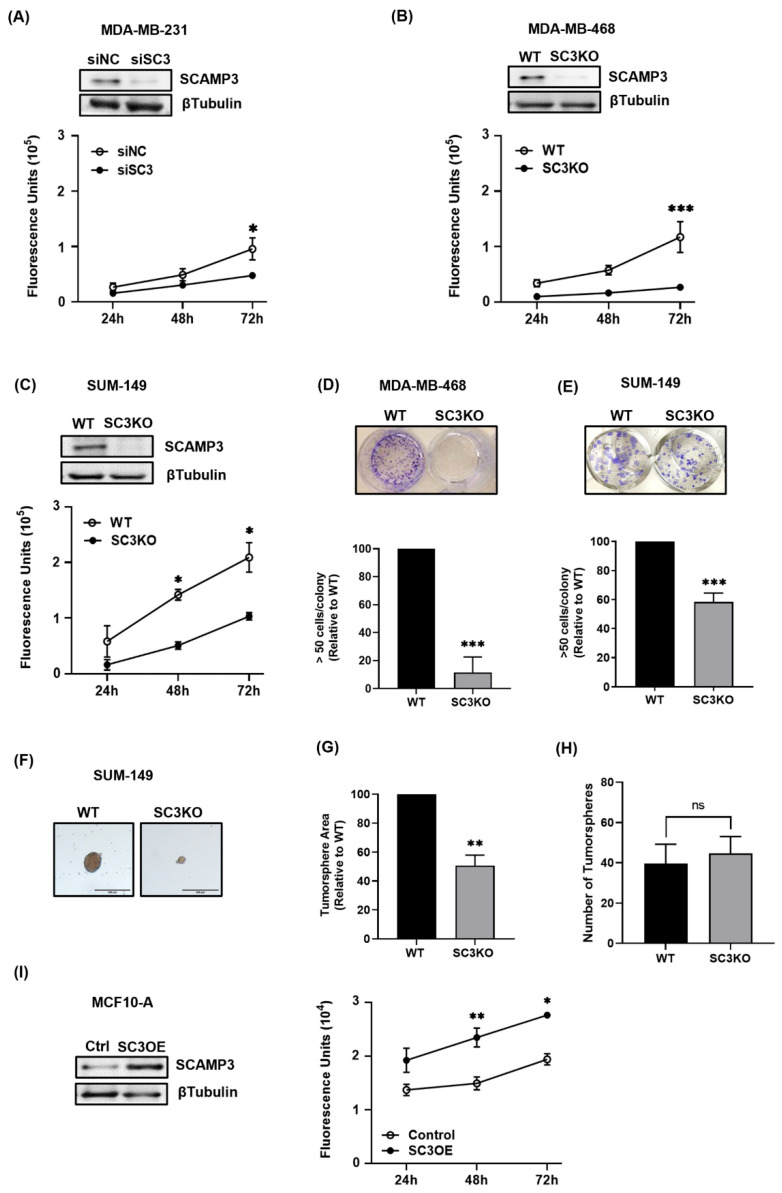
The proliferation of TNBC cells was suppressed after SCAMP3 knockout. (**A**) Immunoblot and proliferation of MDA-MB-231 cells after transfected with SCAMP3 targeting siRNA (siSC3) and nontargeting sequences (siNC). (**B**,**C**) Immunoblotting and proliferation of wild-type (WT) MDA-MB-468 and SUM-149 cells after SCAMP3 gene knockout (SC3KO) using CRISPR/Cas9. WT and SC3KO (**D**) SUM-149 and (**E**) MDA-MB-468 cells were incubated for ten days. The colonies were stained with crystal violet and the relative number of colonies with >50 cells was graphed. Student’s *t*-test; *** *p* ≤ 0.001. WT and SC3KO SUM-149 were allowed to grow in a low-attachment plate for three days to allow them to form tumorspheres. (**F**) Illustrated micrographs represent 5 photos per condition. (**G**) Area and (**H**) number of spheres were quantified using ImageJ. Scale bar = 200 µm. Student’s *t*-test ** *p* < 0.01, ns: not significant. (**I**) Immunoblot and proliferation of non-tumorigenic mammary epithelial cells MCF-10A after transfection with SCAMP3 cDNA (SC3OE). Cell proliferation was examined for three consecutive days using the CyQUANT^®^ NF Cell Proliferation Assay. Two-way ANOVA, * *p* ≤ 0.05; ** *p* < 0.01; *** *p* < 0.001. Data are represented as mean ± SEM. The experiments were carried out at least three times. All Western blot images can be fund at Figure S2.

We previously reported high SCAMP3 expression in invasive ductal carcinoma and inflammatory breast cancer tumors. Moreover, we did not observe protein expression in normal tissues [10]. In recent studies, researchers have reported SCAMP3 as a promoter of cell proliferation in hepatocellular carcinoma, glioma, and melanoma [11,12,14]. We hypothesized that silencing SCAMP3 in TNBC cell lines would decrease proliferation, clonal expansion, and their capacity to form spheres. To test this, we silenced SCAMP3 in MDA-MB-231, MDA-MB-468, and SUM-149 TNBC cells with SCAMP3 targeting siRNA (siSC3) or stable CRISPR-Cas9 vectors (SC3KO) and monitored the effects after 24, 48, and 72 h after transfection. Knockout efficiency was determined by immunoblotting. The proliferation data showed that SCAMP3 silencing significantly decreased the proliferation of all cell lines at 72 h (MDA-MB-231 (siSC3) and SUM-149 SC3KO: 51%; and MDA-MB-468 SC3KO: 33%) compared to control or wild type (WT) cells (Figure 1A–C). Consistent with proliferation results, the colony formation capacity of MDA-MB-468 and SUM-149 cells was impaired in suppressed SCAMP3 cells (Figure 1D,E). We also evaluated whether the reduction in SUM-149 proliferation was translated into a 3D culture. We observed that SC3KO cells formed the smallest tumorspheres but did not affect their quantity (Figure 1F–H).

To validate that SCAMP3 alters malignant cell proliferation, we overexpressed SCAMP3 in non-tumorigenic epithelial mammary cells, MCF-10A. As expected, cells overexpressing SCAMP3 (SC3OE) increased proliferation 48 h after transfection by ~60% compared to controls (Figure 1I).

### 3.2. Silencing SCAMP3 Modulates EGFR Oncogenic Signaling

**Figure 2:**
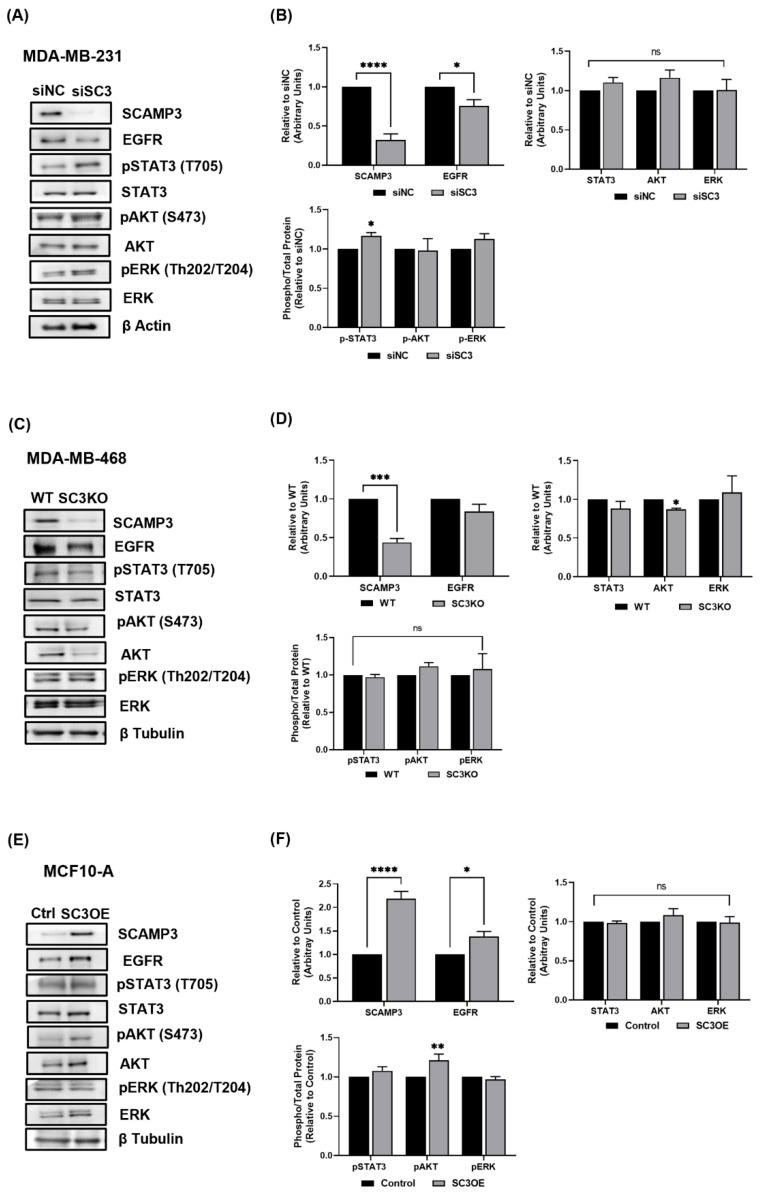
SCAMP3 modulates the EGFR signaling pathway. Immunoblots and densitometry quantification of (**A**,**B**) MDA-MB-231 cells transfected with SCAMP3 targeting siRNA and non-targeting sequence (siNC). (**C**,**D**) MDA-MB-468 WT and SC3 knockout cells. (**E**,**F**) MCF-10A control cells and SC3 overexpressing cells (SC3OE). Lysates were probed using the indicated antibodies. Two-way ANOVA with Bonferroni’s multiple comparison test; * *p* ≤ 0.05, ** *p* < 0.01, *** *p* < 0.001 and **** *p* < 0.0001. Data are represented as mean ± SEM. The experiments were carried out at least three times.

The expression of EGFR was reported in 80% of TNBC, thus, it is considered an attractive target [20]. Furthermore, previous studies have identified SCAMP3 as a target of EGFR through SCAMP3 tyrosine phosphorylation [7,14,21,22]. Therefore, we explored the effects of SCAMP3 silencing on proteins associated with EGFR signaling. Immunoblot data showed that low expression of SCAMP3 decreased EGFR and AKT in MDA-MB-231 cells and MDA-MB-468, respectively (Figure 2A–D). Interestingly, silencing of SCAMP3 increased STAT3 activation at the Tyr 705 residue in MDA-MB-231 cells (Figure 2A,B). According to our hypothesis that SCAMP3 modulates the EGFR pathway, cells overexpressing SCAMP3 showed increased expression of EGFR and increased activation of AKT (Figure 2E,F). We consider it important to note that cells overexpressing MCF-10A SCAMP3 were seeded in their complete growth medium supplemented with EGF when proliferation and protein expression were assessed. These results evidence that SCAMP3 regulates EGFR, AKT, and STAT3 in TNBC.

### 3.3. SCAMP3 Depletion Delays Tumor Cell Proliferation at the Beginning of Tumor Development and Decreases EGFR Activation

**Figure 3:**
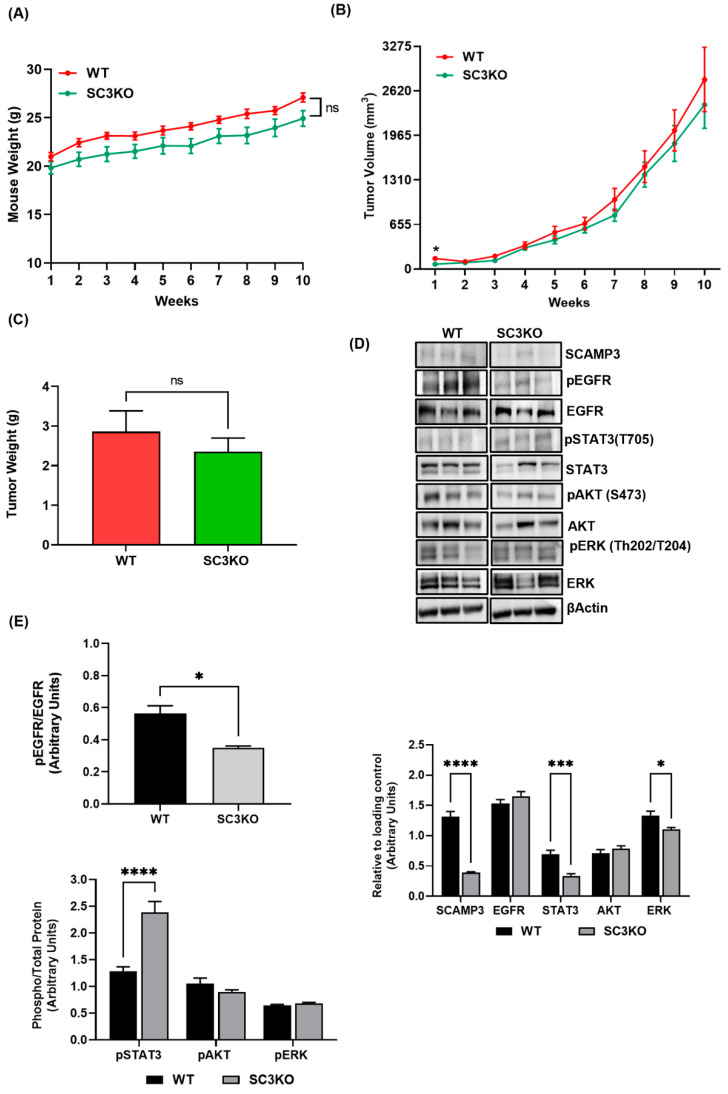
SCAMP3 delayed tumor cell proliferation and decreased p-EGFR. Orthotopic models of breast cancer were generated using SUM-149 cells. (**A**) Body weight of mice injected with wild-type (WT) (*n* = 9) and SCAMP3 knockout cells (SC3KO) (*n* = 9). Two-way ANOVA: non-significant. (**B**) Tumor volume (mm^3^) was monitored for 10 weeks. Comparisons of the average tumor size by group and week were evaluated by unpaired t-test assuming unequal variances; * *p* ≤ 0.05. (**C**) Tumor weights at the end of the study. *t*-test; ns: not significant. (**D**) Immunoblots of SUM-149 tumors. Each lane represents a different animal. Samples are representative of *n* = 8/group. Lysates were probed using indicated antibodies. (**E**) Densitometry quantification analyses of (*n* = 8/group). Two-way ANOVA; * *p* ≤ 0.05, *** *p* < 0.001 and **** *p* < 0.0001; *t*-test. Data are represented as mean ± SEM.

We investigated the role of SCAMP3 in the regulation of TNBC tumor growth. We hypothesized that SCAMP3 depletion would affect tumor development and progression. The absence of SCAMP3 did not adversely affect the health of mice (Figure 3A). Contrary to what we expected, SCAMP3 depletion did not decrease tumor volume. However, a tumor size reduction was observed in week one compared to WT (Figure 3B). No significant reduction in tumor weight was observed at the end of the study (Figure 3C). These observations suggest that the reduction in cell proliferation observed in vitro is not sustained in vivo where other factors in the tumor microenvironment could contribute to tumor growth. We investigated whether EGFR signaling was modulated in SC3KO tumors. SCAMP3 depletion decreased EGFR activation. Interestingly, STAT3 phosphorylation increased in SCAMP3 silenced tumors (Figure 3D,E), consistent with what we observed in MDA-MB-231 cells. STAT3 activation could explain the progression of SC3KO tumors.

### 3.4. SCAMP3 Colocalizes with EGFR after Receptor Activation

Our data indicated that silencing SCAMP3 modulates total expression and activation of EGFR in vitro and in vivo, respectively. Therefore, we sought to investigate the cellular mechanism behind this regulation. EGFR ligand stimulation causes receptor activation, internalization, and trafficking to early endosomes that eventually degrade to lysosomes or are recycled to the cell surface. The internalization of EGFR is differentially regulated by its ligands and several mechanisms that contribute to cancer development [23,24,25,26]. The Castle group showed an interaction of SCAMP3 and EGFR within the cell upon EGF stimulation in murine fibroblasts that overexpress EGFR and HeLa models [7,21]. However, this model has not been explored in cancer.

**Figure 4:**
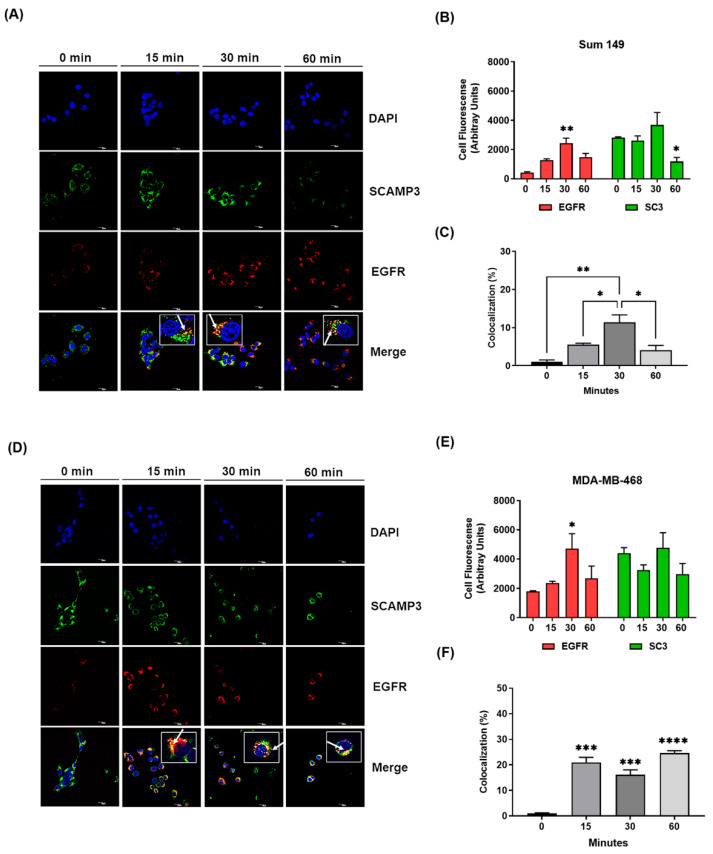
SCAMP3 colocalizes with EGFR after receptor internalization. Cells were stimulated with 10 ng/mL EGF at the indicated time points to evaluate the location of SCAMP3 and EGFR using confocal microscopy. Representative images of the internalization assay and fluorescence quantification of SCAMP3, EGFR, and their colocalization in (**A**–**C**) SUM-149 and (**D**–**F**) MDA-MB-468 cells. The nuclei were stained with DAPI (blue). Secondary antibodies conjugated to Alexa 488 (green) and Alexa 594 (red) were used to detect SCAMP3 and EGFR, respectively. The micrographs were taken at a magnification of 60× using confocal microscopy. The white arrows indicate the colocalization of EGFR and SCAMP3 in the zoom images. The zoom-in of each image is shown in white squares and each has equal dimensions. Scale bar = 20 µm. Total cell fluorescence and colocalization area analyses were performed in 20 cells from three experiments using Image J. Colocalization was calculated as the ratio of the colocalization fluorescence area to the total cell fluorescence area. One way or two-way ANOVA; * *p* ≤ 0.05, ** *p* < 0.01, *** *p* < 0.001, **** *p* < 0.0001. Data are represented as mean ± SEM.

We performed internalization assays of antibodies to assess whether the model proposed by others would be validated in our TNBC models. As illustrated in Figure 4A, SCAMP3 localizes to punctate structures distributed throughout the cytoplasm and shows accumulation in the perinuclear area in SUM-149 cells (EGFR^+^). Although no redistribution of SCAMP3 was observed after ligand stimulation, EGFR accumulates in the perinucleus after 15 min, showing colocalization with SCAMP3 in this area. We observed a higher expression of EGFR and colocalization with SCAMP3 at 30 min (Figure 4B,C). At one hour, receptor staining was detected in the cytoplasm and less colocalization with SCAMP3 was observed. Interestingly, SCAMP3 expression also decreased. Contrary to what we observed in SUM-149 cells after EGF stimulation, SCAMP3 is redistributed from punctate cytoplasmic structures to the perinuclear area and nucleus to colocalize with EGFR in MDA-MB-468 cells (EGFR^+++^), suggesting a possible role for SCAMP3 in receptor nuclear translocation (Figure 4D). Although the highest expression of EGFR was observed at 30 min (Figure 4E), similar to SUM-149, the expression levels of SCAMP3 and colocalization with EGFR were constant throughout time (Figure 4F). These results suggest degradation of SCAMP3 and EGFR 60 min after receptor stimulation in SUM-149 cells. In summary, SCAMP3 interacts directly with EGFR after internalization of the receptor and contributes to receptor trafficking and, possibly, to its degradation.

### 3.5. SCAMP3 Regulates the Migration and Invasion of TNBC Cells

**Figure 5:**
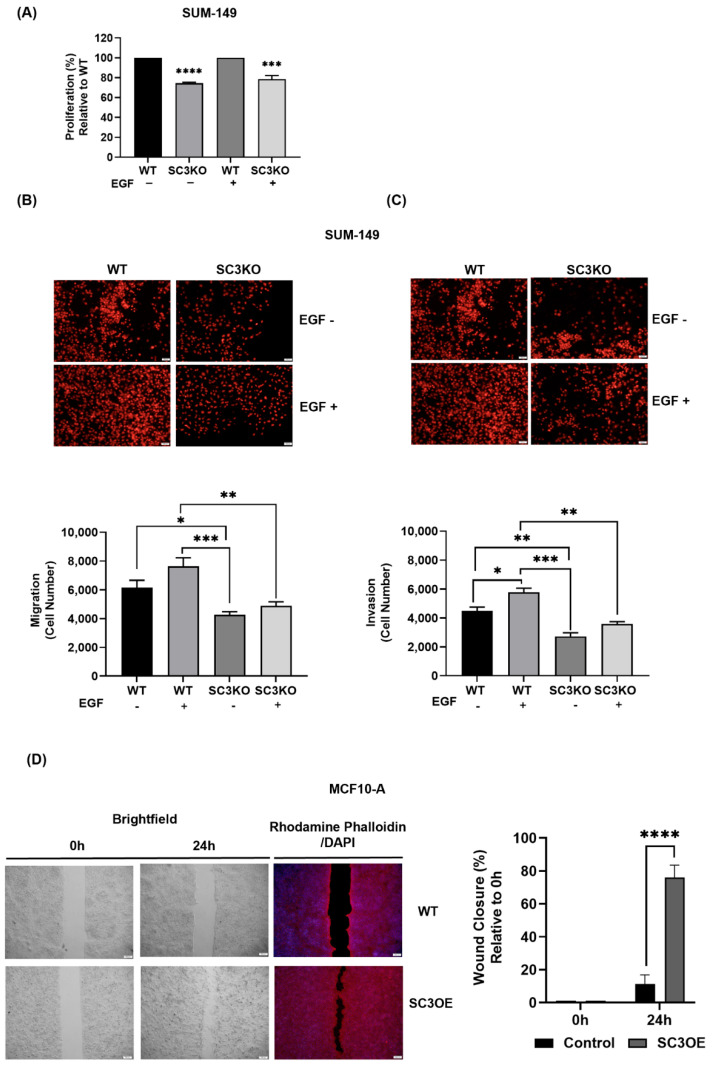
SCAMP3 modulates TNBC cell proliferation and motility through EGFR. (**A**) SUM-149 WT and SC3KO cells were stimulated with 10ng/mL of EGF for 30 min and proliferation was measured after 72 h using the Cell Proliferation Kit I (MTT). Data are expressed relative to WT without (EGF-) or after stimulation (EGF+). One-way ANOVA; *** *p* < 0.001 and **** *p* < 0.0001. EGF-stimulated SUM-149 WT stimulated with EGF and SC3KO were incubated in migration and Matrigel invasion chambers for 24 h to assess (**B**) migration and (**C**) invasion ability of cells. The nuclei of the migrating and invading cells were stained with PI. Illustrations represent 14 micrographs per condition at a magnification of 200×; scale = 100 μm. Cells were counted using ImageJ. Two-way ANOVA; * *p* ≤ 0.05, ** *p* < 0.01, *** *p* ≤ 0.001. (**D**) Wound healing assay of MCF-10A control and SC3OE cells using silicone insert Ibidi^®^ plates after 24 h. The width of the wound was determined by measuring the distance between the edges of the wound. The nuclei were stained with DAPI (blue) and the actin cytoskeleton was stained with Rhodamine Phalloidin (red). Micrographs were taken at a magnification of 400×; scale = 200 μm. Data are expressed as percent relative to 0 h and analyzed relative to control cells. *t*-test: **** *p* ≤ 0.0001. Data are represented as mean ± SEM. The experiments were carried out at least three times.

Since we observed a direct and stronger association of SCAMP3 and EGFR 30 min after receptor activation, we sought to investigate the effects of silencing SCAMP3 after EGF stimulation on cell proliferation. We stimulated SUM-149 WT and SC3KO cells with EGF for 30 min and evaluated cell proliferation. Proliferation was still reduced in SC3KO cells at 72 h when EGFR is activated compared to WT (Figure 5A) according to what we observed in Figure 1C.

SCAMP3 has been identified to promote the proliferation of glioma and melanoma; however, no evident correlation has been found between SCAMP3 and its regulation of cell migration and invasion [11,14]. Only Zhang et al. correlated high SCAMP3 expression with vascular invasion in hepatocellular carcinoma [11]. Therefore, we investigated whether the association of EGFR and SCAMP3 promotes cell motility and how it is affected in SC3KO SUM-149 cells. To ensure that the effect of SCAMP3 silencing in SUM-149 was not due to a reduction in proliferation, we performed migration and invasion assays after 24 h of EGF stimulation. Depletion reduced cell migration and invasion. Importantly, EGFR activation did not abrogate these effects (Figure 5B,C). Finally, we investigated whether SCAMP3 overexpression promotes non-cancerous cell migration. The results showed an increased capacity of cells to close the wound, validating that SCAMP3 plays a key role in motility (Figure 5D).

### 3.6. SCAMP3 Regulates EGFR through Degradation and Modulates AKT, ERK, and STAT3

**Figure 6:**
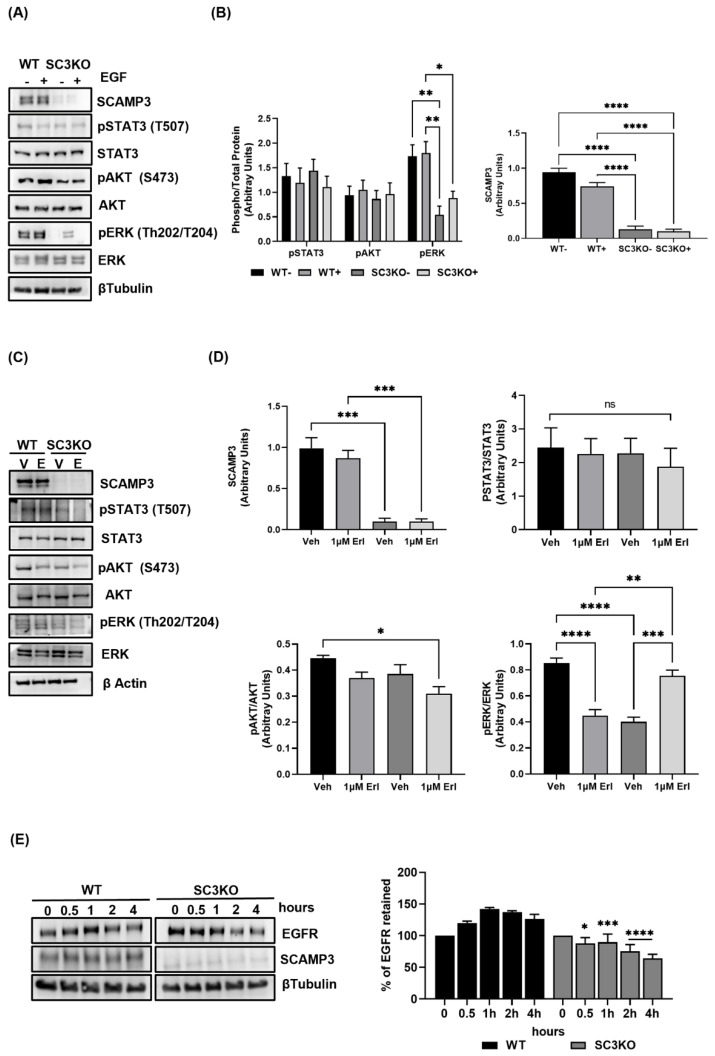
SCAMP3 regulates EGFR signaling by degradation of the receptor. (**A**,**B**) Immunoblots and densitometry quantification of SUM-149 WT or SC3KO cells after stimulation with 10 ng/mL EGF for 30 min. Lysates were probed using the indicated antibodies. Two-way ANOVA; * *p* ≤ 0.05, ** *p* < 0.01, **** *p* < 0.0001. (**C**,**D**) Immunoblots and densitometry quantification analyses of SUM-149 WT or SC3KO lysates after cells treated with 1 µM erlotinib for 72 h. One-way ANOVA; * *p* ≤ 0.05, ** *p* < 0.01, *** *p* < 0.001, **** *p* < 0.0001, ns = not significant. (**E**) Serum starved WT and SC3KO SUM-149 cells were treated with 100μM cycloheximide for 1 h before stimulation with 10 ng/mL of EGF. Lysates were obtained after each time point shown and immunoblotted using SCAMP3 and EGFR antibodies. After densitometry analysis, the EGFR intensities data were normalized to β-tubulin and correlated to 0 h. The plotted data represent the residual EGFR. *t*-test, * p ≤ 0.05, *** *p* < 0.001 and **** *p* < 0.0001. Data are represented as mean ± SEM. The experiments were carried out at least three times.

We showed that EGFR activation did not alter the effect of decreasing SCAMP3 in our isogenic models. To assess how EGFR signaling is affected by EGF stimulation, we used our SUM-149 WT and SCAMP3 knockout models. Immunoblots showed that, contrary to what we observed in MDA-MB-231 and MDA-MB-468 (Figure 2A,B), SCAMP3 knockout in SUM-149 abolished ERK1/2. Interestingly, EGFR activation resulted in slightly increased ERK phosphorylation compared to unstimulated SC3KO cells, but less phosphorylated than WT stimulated cells (Figure 6A). As expected, ERK activation does not depend exclusively on the interaction of SCAMP3 and EGFR. However, based on our results, it is possible that SCAMP3 is directly associated with ERK activation in these cells. Thus, we inhibited EGFR activation with the tyrosine kinase inhibitor (TKI), erlotinib. Our group and others have published that inhibition of EGFR by erlotinib decreases ERK activation in SUM-149 cells [27,28]. We validated that SCAMP3 depletion decreases ERK activation. Furthermore, erlotinib treatment reduced ERK phosphorylation in SCAMP3-expressing cells, as expected, but was not affected in depleted cells. However, ERK phosphorylation increased compared to SCAMP3 depleted cells treated with vehicle. Interestingly, the treatment decreased AKT phosphorylation in the absence of SCAMP3. Furthermore, inhibition of EGFR did not affect STAT3. This may suggest that SCAMP3 is involved in the regulation of the EGFR, AKT, ERK, and STAT3 pathways. Therefore, we sought to investigate the molecular mechanism behind the regulation of EGFR by SCAMP3.

Castle et al. proposed two models to define the SCAMP3 function to inhibit EGFR degradation. They suggested that after internalization, a portion of EGFR is sorted into lysosomes for degradation through ubiquitination and ESCRT (endosomal sorting complex required for transport) dependent [7]. However, three years later, using a different model [Baby Hamster Kidney fibroblasts (BHK cells)] previously used, they published that instead of inhibiting EGFR degradation, SCAMP3 promoted it [6]. Therefore, we examined the impact of SCAMP3 silencing on receptor degradation after stimulation. We used WT and SCAMP3 knockout SUM-149 cells in a time-course experiment from 0–4 h. These cells were serum-starved, treated with cycloheximide to inhibit translation, and then stimulated with EGF. Upon stimulation, the percentage of EGFR retained in the cell decreased in SCAMP3 knockout cells as early as 30 min showing an acceleration in the degradation kinetics of EGFR (Figure 6C). In summary, depletion of SCAMP3 decreases ERK phosphorylation independently of its interaction with EGFR. Concurrently, the reduction of AKT phosphorylation depends on the downregulation of both. Furthermore, the absence of SCAMP3 accelerates the degradation of EGFR.

### 3.7. Clinical Relevance of SCAMP3 in Breast Cancer Patients

**Figure 7:**
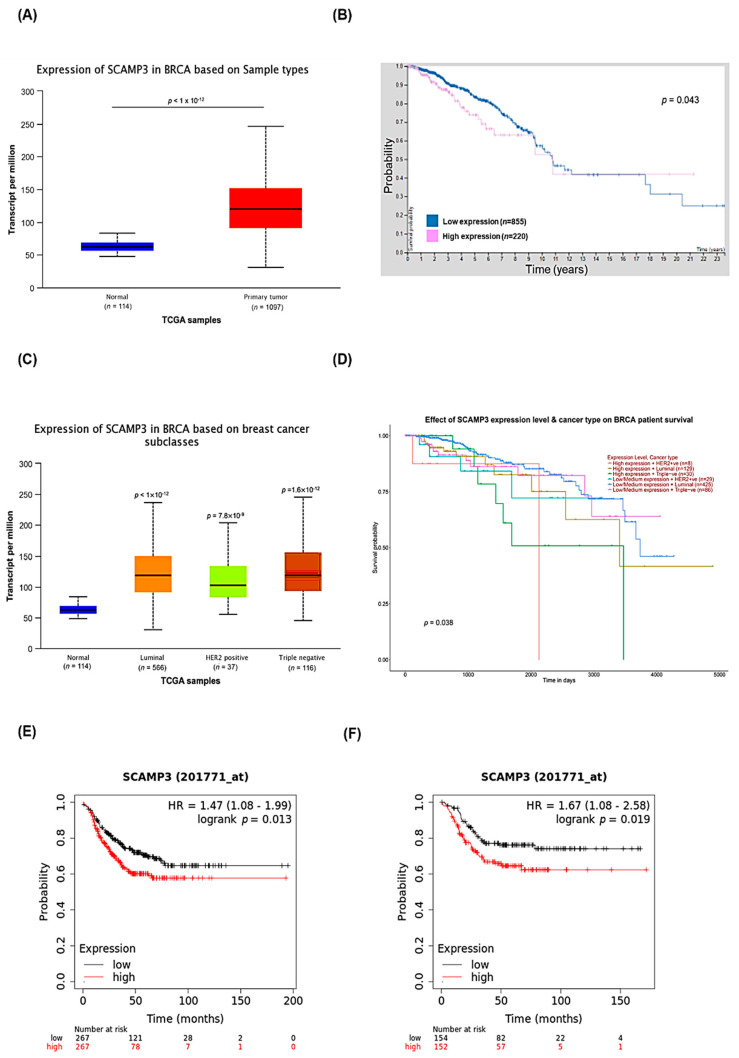
SCAMP3 expression analyses from the TCGA database. (**A**) UALCAN portal analysis comparing SCAMP3 expression between normal and breast cancer tumors. (**B**) Probability of survival between breast cancer patients with high and low SCAMP3 expression using The Human Protein Atlas portal. (**C**) Expression of SCAMP3 in different subtypes of breast cancer. (**D**) Probability of survival between breast cancer patients with different subtypes and low to high levels of SCAMP3 expression. Green line = SCAMP3 high expression (TNBC). (**E**) Probability of relapse-free survival (RFS) between TNBC patients with high and low SCAMP3 expression using Kaplan–Meier Plotter portal. (**F**) Probability of distant metastasis-free survival (DMFS) between patients with TNBC with high and low SCAMP3 expression using Kaplan–Meier Plotter portal.

Since SCAMP3 is overexpressed in several cancers, we evaluated its expression in breast cancer patients included in the TCGA database using the UALCAN and Human Protein Atlas portals [13,14,15,17,29,30,31]. The analysis showed SCAMP3 overexpression in primary breast tumors (*n* = 1097) compared to normal tissues (*n* = 114) (Figure 7A). Consistent with this observation, breast cancer patients with SCAMP3 overexpressed tumors showed a decreased survival probability (*n* = 220). Five-year survival for the group with higher expression of SCAMP3 was 74% compared to 83% for patients with lower expression levels (*p* = 0.043) (Figure 7B).

We also evaluated the expression of SCAMP3 in breast cancer subclasses. Remarkably, we did not observe differences between subtypes (Figure 7C). Interestingly, patients with TNBC with elevated SCAMP3 have a decreased survival probability compared to patients with luminal tumors (*p* = 0.038) (Figure 7D). We analyzed data on relapse-free survival (RFS) and distant metastasis-free survival (DMFS) in TNBC using the Kaplan–Meier Plotter database. TNBC patients with high expression of SCAMP3 have decreased RFS (*p* = 0.013) (Figure 7E) and DMFS probability (*p* = 0.019) (Figure 7F) than patients with low SCAMP3 tumors.

### 3.8. SCAMP3 Regulates the Expression of Genes Associated with EGFR and PDGFR Signaling

**Figure 8:**
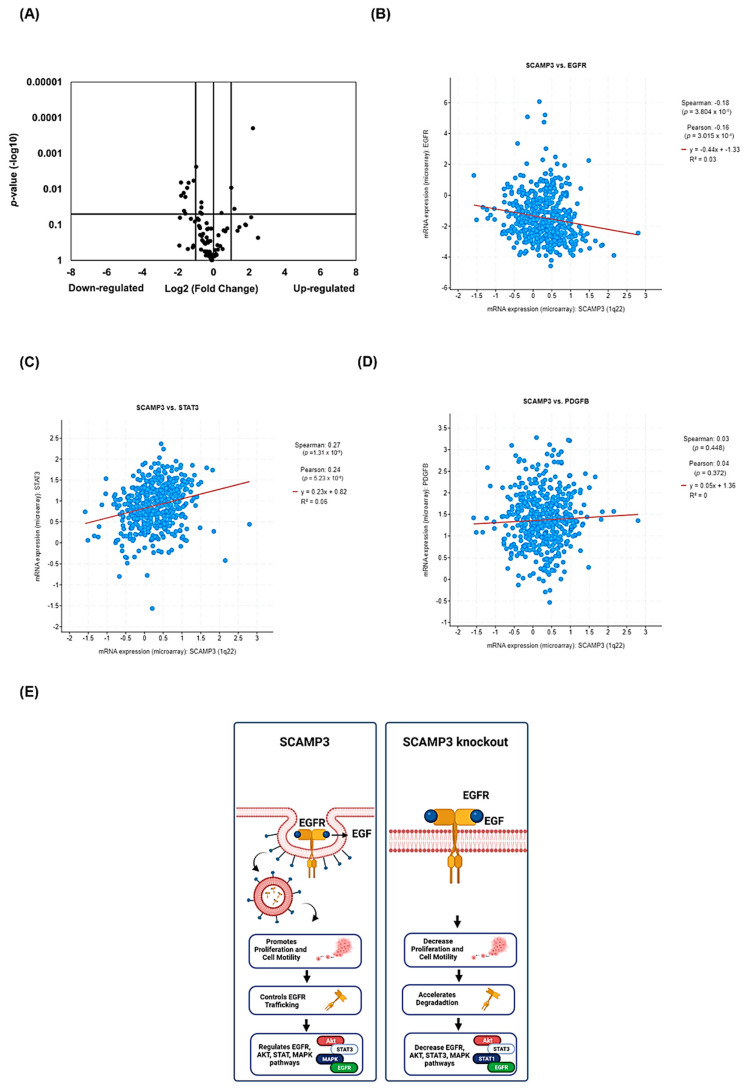
Regulation of gene expression by SCAMP3 in breast cancer. (**A**) RT^2^ PCR arrays were performed to profile the expression of EGF/PDGF signaling pathway-specific genes in three different tumors per group. *ACTB*, *B2M*, *GAPDH*, *HPRT1*, and *RPLP0* were used as reference genes. The Volcano plot shows the effects on gene expression analyzed at −2.0 ≥ 2.0 log_2_-fold change (vertical lines). Statistically significant (*p* ≤ 0.05) genes are above the horizontal black line. Correlation of the expression of the SCAMP3 and (**B**) EGFR, (**C**) STAT3, or (**D**) PDGF genes in breast cancer. BioPortal web server: Breast Invasive Carcinoma (TCGA, Firehouse Legacy). (**E**) Summary of our findings. The left panel shows the interaction of EGFR and SCAMP3 within the cell after activation of EGFR in WT cells and its effects on cell proliferation, motility, and modulation of EGFR, AKT, and ERK. The right panel shows our findings in our SCAMP3 depletion models. EGFR depletion decreased cell proliferation, migration, and invasion. Moreover, accelerated EGFR degradation and modulated EGFR, AKT, ERK, and STAT3.

We investigated the function of SCAMP3 as a regulator of genes associated with the EGFR pathway. We also explored a possible association of SCAMP3 with the PDGFR pathway, which has not yet been studied. We performed EGF/PDGF Signaling Pathway RT^2^ Profiler PCR arrays to conduct these experiments using SUM-149 tumor samples. As shown in Figure 8A and Table 1, SCAMP3 depletion reduced the expression of 100% of statistically different genes (*p* ≤ 0.05) that displayed −2.0 ≥ 2.0 fold up-or down-regulation change. Depletion of SCAMP3 decreased the expression of *AKT1* and *AKT2* (−2.6 f.ch.), which encode two of the three members of the human AKT serine-threonine protein kinase family. Interestingly, we did not observe decreased expression of total AKT protein in tumors evaluated with an anti-AKT antibody that detects endogenous levels of total AKT1, AKT2, and AKT3, suggesting that AKT transcript levels do not influence protein translation. We also observed down-regulation of *FN1* transcript (−3.1 f.ch.), which encodes preprotein fibronectin, a proteolytically processed glycoprotein involved in cell adhesion and migration processes, wound healing, and metastasis [32]. Cell cycle regulator *CCND1* (cyclin D1) was also down-regulated (−3.2 f.ch.) in SCAMP3 depleted tissues. Other groups have published an accumulation of SCAMP3 melanoma knockout cells in the S and G2/M cell cycle phases and arrest in G1 in HCC SCAMP3 depleted cells [11,12]. The *GSK3A* and *GSK3B* genes were deregulated by −3.19 f.ch. and −1.5 f.ch., respectively. These genes codify the multifunctional Ser/Thr kinase glycogen synthase kinase 3α and β isoforms, which are components of the EGFR/AKT pathway and are associated with tumor development, angiogenesis, metastasis, and drug resistance [33].

We expected that SCAMP3 depletion in SUM-149-derived tumors would significantly affect ERK pathway proteins, as we observed in our in vitro studies. However, we identified the deregulation of several MAPK genes in the PCR array assay. The gene expression analysis identified significant down-regulation of *BRAF* (−3.0 f.ch.), *KRAS* (−2.1 f.ch.), *MAP2K1* (Mitogen-Activated Protein Kinase Kinase 1 or MEK1) (−1.9 f.ch.), and *MAP2K7* (Mitogen-Activated Protein Kinase Kinase 7) (−2.0 f.ch.). Furthermore, the gene expression of *MAP3K2* (Mitogen-Activated Protein Kinase Kinase Kinase 2) and *MAPK8* (Mitogen-Activated Protein Kinase 8 or JNK) decreased by −1.6 and −1.5 f.ch., respectively (Table 1). *MAP2K1* (−2.2 f.ch.) and *MAPK3* (−1.3 f.ch.) levels were also reduced in SCAMP3 depleted SUM-149 cells (Supplementary Figure S1A,B and Table S1). The Ras/MAPK pathway is a major component of oncogenesis activity in TNBC [34]. Ras family members are small GTPases activated by external stimuli, including the activation of tyrosine kinase receptors. Ras facilitates the activation of Raf, which starts a kinase cascade through MEK and ERK, resulting in nuclear translocation of ERK and activation of transcription factors such as *ELK1*, which we identified as down-regulated (−1.6 f.ch.) (Table 1) [35].

Interestingly, SCAMP3 depletion decreased the expression of *PDGFB* (Platelet-Derived Growth Factor Subunit β) (−2.6 f.ch.) and *NCK2* (Non-catalytic region of tyrosine kinase (NCK) adaptor protein 2), which is recruited to activate tyrosine kinase receptors such as EGFR and PDGFR [36]. *PDGFB* was also down-regulated in cells (−3.2 f.ch.) (Supplementary Table S1). We hypothesized that *STAT3* transcript could be deregulated in SCAMP3 depleted tissues because we observed a downregulation of total STAT3 protein. However, STAT3 mRNA was not significantly affected (Supplementary Figure S1A). Instead, we observed a down-regulation of *STAT5A* in tumors (−7.1 f.ch.) (Table 1) and cells (−3.6 f.ch.) (Supplementary Table S1). The protein encoded by this gene is a member of the STAT family of transcription factors recently identified as a resistance inducer to doxorubicin [37].

Next, we investigated the expression patterns of EGFR, STAT3, and PDGF in the TCGA dataset. As shown in (Figure 8B), the correlation analysis demonstrated that SCAMP3 expression displayed a weakly negative correlation with EGFR (Pearson CC = −0.16; *p* = 3.015 × 10^−4^) (Figure 8C), a weakly positive correlation with STAT3 (Pearson CC = 0.24; *p* = 5.23 × 10^−8^) and no association with *PDGFB* (Pearson CC = 0.04; *p* = 0.372) (Figure 8D).

We summarized our findings in Figure 8E. The left panel shows the interaction of EGFR and SCAMP3 within the cell after receptor activation in WT cells and its effects on cell proliferation, motility, and modulation of EGFR, AKT, and ERK. The right panel shows our findings in our SCAMP3 depletion models. EGFR depletion decreased cell proliferation, migration, and invasion. Moreover, accelerated EGFR degradation and modulated AKT, ERK, and STAT3.

## 4. Discussion

The potential role of SCAMP3 in breast cancer remains unexplored. This study investigated the role of SCAMP3 in promoting TNBC cell response and tumor progression. SCAMP3 is an integral membrane protein component of the eukaryotic cell surface recycling system [5,6]. It has been found to be overexpressed and associated with poor overall survival in glioma, hepatocellular and pancreatic adenocarcinomas [11,14,15]. Previously, we published a novel study demonstrating an increased expression of SCAMP3 in inflammatory breast cancer (IBC) and invasive ductal carcinoma (IDC) tumor tissues. We confirmed the expression of SCAMP3 in invasive ductal carcinoma patient samples; however, we did not observe its expression in non-cancerous breast tissues [10]. Based on our previous findings and the limited number of studies that evaluate the role of SCAMP3 in cancer, we sought to investigate the role of SCAMP3 in TNBC and the molecular mechanism behind its function. Here, we reveal numerous novel aspects of the function of SCAMP3 as a regulator of oncogenesis through several mechanisms.

We showed that SCAMP3 depletion decreased the proliferation of our three TNBC cell models and their ability to form colonies and tumorspheres. At the same time, its overexpression promoted the proliferation of non-cancerous mammary cells. Although SCAMP3 has been associated with cancer cell proliferation, only one study correlated this protein with invasion [11]. Here, we demonstrate that depletion of SCAMP3 decreased migration and invasion of TNBC cells while overexpression of SCAMP3 promoted migration of non-cancerous cells. Our results contrast with recent reports from other groups in other cancer types, which show that SCAMP3 depletion did not affect cancer cell motility [12,14].

Given that our results suggested that SCAMP3 knockout would decrease tumor growth, we developed breast cancer xenograft models using WT and SCAMP3 knockout cells. Contrary to our hypothesis, SCAMP3 depletion only reduced tumor volume in the first week of the study. Furthermore, we identified an increase in the variation in tumor size during the last three weeks of the study compared to WT. Although unexpected, these results are novel and could suggest two possible mechanisms of action of SCAMP3. First, the lack of SCAMP3 delays the proliferation of tumor-initiating cells that affects the initial stage of tumor development. Second, SCAMP3 depletion does not affect primary tumor growth, but it might regulate metastasis.

Breast cancer stem cells (BCSCs) are defined as a limited number of tumor-initiating cells capable of self-renewal and differentiation into heterogeneous populations of BC cells [38]. The BCSC subpopulation is more enriched in TNBC cells and tumor tissues than in other BC subtypes, contributing to the development of resistance to chemotherapy, metastasis, and a poor prognosis associated with this subtype [38,39]. Furthermore, EGFR/STAT3 pathway promotes and maintains cancer stemness [40,41]. Aberrant activation of STAT3 also increases the expression levels of pluripotency transcription factors octamer-binding transcription factor 4 (Oct-4) and c-Myc, which regulate stem-mediated resistance to doxorubicin in TNBC [42]. High expression of pSTAT3 also has been found required for TN-BCSC proliferation [43]. Using patient-derived xenograft models (PDX) from TNBC, a group of researchers showed that inhibition of EGFR activation blocked circulating CSCs and lung metastasis [44]. Recently, Ghosh et al. identified an elevated expression of SCAMP3 in glioblastoma multiforme CSCs [45]. However, its role in stemness maintenance was not addressed. Here, we show that SCAMP3 silencing decreased EGFR activation and increased pSTAT3 in tumors. Thus, more studies are needed to explore the role of SCAMP3 in BCSC. Furthermore, studies should be carried out to investigate whether the tumor microenvironment negatively influences the capacity of SCAMP3 depleted cancer cells to alter tumor progression.

The second mechanism we propose is that SCAMP3 depletion could be affecting metastasis as we observed decreased migration and invasion of SUM-149 cells. The SUM-149 cell line is a well-studied model of TN-inflammatory breast cancer. IBC is a rare, aggressive, and deadliest type of breast cancer characterized by its mechanism of invasion and metastasis. The lethality of IBC is due to its ability to invade the vascular and lymphatic systems through the generation of emboli and the development of subsequent metastases [46]. We published an increase in SCAMP3 expression in the hallmark emboli structure and in lymphatic vessels of IBC tumor samples [10]. Therefore, it is necessary to explore the role of SCAMP3 in this type of cancer and how it is associated with IBC metastasis.

SCAMP3 acts as a regulator of EGFR trafficking within endosomal membranes, enhancing receptor recycling and negatively regulating its degradation [7]. Aoh et al. showed the colocalization of internalized EGFR and SCAMP3 in the perinuclear puncta, which corresponds to early endosomes [7]. We validated that SCAMP3 contributes to EGFR internalization and there exists a direct interaction between both proteins in the cytoplasm and the perinucleus in EGFR+ cells after receptor activation. Interestingly, we observed a redistribution of SCAMP3 from the cytoplasm to the perinuclear area and the nucleus in the highly metastatic model of TNBC. These events might suggest that SCAMP3 has a possible role in receptor nuclear transport, which could be explored in further studies.

Castle’s laboratory reported contradictory results addressing the role of SCAMP3 in EGFR degradation. First, they reported that SCAMP3 knockdown promotes EGFR degradation. Later, they published that SCAMP3 depletion inhibits it [6,7]. Here, we demonstrate that SCAMP3 knockout accelerates EGFR degradation. Furthermore, internalization assays showed decreased expression of SCAMP3 along with decreased EGFR one hour after receptor activation, suggesting degradation of both proteins. Our findings support Castle’s first study and open the door to the possibility that SCAMP3 and EGFR degrade in the lysosome.

Some studies have demonstrated that activation of wild-type or mutated EGFR cancer cells stimulates SCAMP3 phosphorylation, promoting the interaction of both proteins [7,21,22]. Due to this association between SCAMP3 and EGFR, recent studies have focused on the EGFR signaling pathway. Recently, other groups have identified a direct interaction of SCAMP3 with tumor suppressor WWOX (WW domain-containing oxidoreductase), the modulation of mTOR signaling, and ERK [14,22,47,48]. Interestingly, Venugopalan et al. demonstrated that SCAMP3 acts as a tumor suppressor in lung adenocarcinoma by modulating ERK, rather than what had been observed in other types of cancer, uncovering its dual role [11,14,22].

To our surprise, SCAMP3 regulates multiple pathways other than EGFR. SCAMP3 depletion decreased total AKT in the highly metastatic and EGFR overexpressing (EGFR^+++^) model of TNBC. Furthermore, SCAMP3 overexpression increased the total activation of EGFR and AKT. In the SUM-149 cell line, SCAMP3 decreased ERK activation. Previously, we published that erlotinib decreased ERK phosphorylation but did not affect AKT activation in SUM-149 cells [27]. In this study, we validated our results and found that inhibition of EGFR and SCAMP3 depletion decreased the phosphorylation of AKT. Therefore, we can conclude that AKT activation depends on the association of EGFR/SCAMP3 and ERK phosphorylation depends on SCAMP3. Furthermore, SCAMP3 depletion decreased EGFR but increased STAT3 activation in MDA-MB-231 cells (EGFR^+^) and SUM-149 tumors. Recently, Song et al. documented a reduction in MDA-MB-231 tumors regulated by the EGFR/JAK/STAT3 pathway [49]. We expected an increase in STAT3 phosphorylation in SCAMP3 SUM-149 depleted cells treated with vehicle and the contrary with erlotinib treatment. However, STAT3 phosphorylation was not affected. These results may support our hypothesis that STAT3 affects tumor development through modulation of BCSC or tumor microenvironment independently of EGFR/SCAMP3.

In recent years, studies on the formation of EGFR heterodimers with family partners, or other RTKs, respectively to promote cancer or drug resistance through multiple pathways, including PI3K/AKT/mTOR, Ras/MEK/ERK, and JAK/STAT have been conducted [50,51,52,53,54,55,56,57]. Several STAT3 targeting strategies use direct STAT3 inhibitors, EGFR inhibitors, and small molecules that affect STAT3 trafficking to prevent STAT3 phosphorylation and transcriptional activation [42]. The crosstalk between the PI3K/AKT/mTOR and JAK/STAT pathways has been identified in TNBC as responsible for resistance to mTOR inhibition [58]. Furthermore, EGFR feedback activation after STAT3 inhibition is the cause of resistance to therapy in pancreatic cancer [59]. STAT3 also increases the survival of EGFR+ cancer stem cells in colorectal cancer [40]. Furthermore, the activation of EGFR in lung cancer cells activated AKT by recycling EGFR to the membrane in gefitinib-resistant cells [60]. Consequently, multiple targeting of these pathways has become a promising therapeutic approach to cancer [61]. EGFR targeting drugs show signs of success in a limited number of TNBC patients due to intrinsic or acquired resistance [62]. The potential role of SCAMP3 down-regulation needs to be further explored in breast cancer patients with resistance to TKI. Targeting SCAMP3 in combination with EGFR inhibitors, interacting RTKs, AKT, ERK, or STAT3 can lead to a better clinical outcome in patients.

We also evaluated the PDGF pathway since EGFR and PDGFR form heterodimers and share common substrates [55]. Recent studies found that SUM-149 BRCA1 mutated cells produce high levels of *PDG-BB* mRNA and inhibition of receptor induces cell apoptosis [63]. Here, we demonstrate the down-regulation of *PDGFB* gene expression in SC3 depleted cells. However, no correlation exists between SCAMP3 and PDGF using TCGA data analysis in invasive breast cancer samples. The knockout of SCAMP3 also impaired the expression of the *STAT5A* gene. STAT5α is a member of the STAT family of transcription factors and is an effector protein downstream of EGFR. The STAT5/JNK pathway has been identified as a modulator of the antineoplastic effects of TKI and has been associated with resistance to treatment [64,65]. Additional experiments are needed to define the relationship between SCAMP3 and PDGF signaling.

It is important to note that *SCAMP3* is highly expressed in breast cancer and patients showed reduced survival. In addition, TNBC patients with high SCAMP3 expression showed less probability to survive than those with luminal tumors. Finally, high expression of SCAMP3 in patients with TNBC was associated with low relapse-free survival (RFS) and distant metastasis-free survival (DMFS).

Consistent with the findings that identified SCAMP3 as a tumor-promoting protein, our results indicated that SCAMP3 plays a significant role in the oncogenesis and progression of TNBC through the regulation of EGFR degradation and other multiple pathways. In this report, we find evidence that SCAMP3 serves as a targetable marker. Given that inhibition of SCAMP3 efficiently promotes cell death, decreases cell migration, and invasion, it indicates the potential to tailor specific therapies to breast cancer patients. Furthermore, our novel results open new opportunities for further research to explore the effectiveness of targeting SCAMP3 in combination with other agents. Therefore, in general, this study uncovers a targetable SCAMP3-EGFR-AKT-ERK-STAT3 pathway with biological and therapeutic significance for TNBC.

## 5. Conclusions

Through our findings, we demonstrate, for the first time, the role of SCAMP3 in promoting breast cancer proliferation, migration, and invasion, through negative regulation of EGFR degradation, as well as AKT, ERK, and STAT signaling pathways. Furthermore, we showed that SCAMP3 regulates the expression of *PDGF* and associated genes. The present study provides novel mechanistic insights into the therapeutic potential of targeting SCAMP3 in breast cancer and reveals that the combination of EGFR inhibitors and SCAMP3 depletion displays promising activity to enhance therapeutic responsiveness in patients with resistance to EGFR TKI.

## Figures and Tables

**Table 1:** In vivo effects of SCAMP3 depletion on the expression of *EGF/PDGF* signaling pathway genes.

Symbol	Name	Fold * Change	*p*-Value *
***AKT1***	V-akt murine thymoma viral oncogene homolog 1	−2.6	0.009
***AKT2***	V-akt murine thymoma viral oncogene homolog 2	−2.6	0.02
***AKT3***	V-akt murine thymoma viral oncogene homolog 3 (protein kinase B, gamma)	−1.8	0.05
***ATF1***	Activating transcription factor 1	−1.8	0.03
***ATF2***	Activating transcription factor 2	−1.5	0.03
***BCAR1***	Breast cancer anti-estrogen resistance 1	−1.9	0.02
***BRAF***	V-raf murine sarcoma viral oncogene homolog B1	−3.0	0.03
***CCND1***	Cyclin D1	−3.2	0.006
***ELK1***	ELK1, member of ETS oncogene family	−1.6	0.02
***EPS8***	Epidermal growth factor receptor pathway substrate 8	−1.6	0.01
***FN1***	Fibronectin 1	−3.1	0.04
***GSK3A***	Glycogen synthase kinase 3 alpha	−3.2	0.001
***GSK3B***	Glycogen synthase kinase 3 beta	−1.5	0.003
***KRAS***	V-Ki-ras2 Kirsten rat sarcoma viral oncogene homolog	−2.1	0.00002
***LTA***	Lymphotoxin alpha (TNF superfamily, member 1)	−1.8	0.02
***MAP2K1***	Mitogen-activated protein kinase kinase 1	−1.9	0.001
***MAP2K7***	Mitogen-activated protein kinase kinase 7	−2.0	0.04
***MAP3K2***	Mitogen-activated protein kinase kinase kinase 2	−1.6	0.02
***MAPK8***	Mitogen-activated protein kinase 8	−1.5	0.01
***MKNK1***	MAP kinase interacting serine/threonine kinase 1	−1.6	0.001
***NCK2***	NCK adaptor protein 2	−2.3	0.005
***NRAS***	Neuroblastoma RAS viral (v-ras) oncogene homolog	−1.5	0.03
***PDGFB***	Platelet-derived growth factor beta polypeptide	−2.6	0.002
***PDPK1***	3-phosphoinositide dependent protein kinase-1	−1.5	0.02
***PPP2CA***	Protein phosphatase 2, catalytic subunit, alpha isozyme	1.8	0.03
***RASA1***	RAS p21 protein activator (GTPase activating protein) 1	−1.6	0.003
***STAT5A***	Signal transducer and activator of transcription 5A	−7.1	0.003

* Table shows genes up- and down-regulated ≥1.5-fold change and *p* ≤ 0.05.

## Data Availability

Not applicable.

## Supplementary Materials

The following supporting information can be downloaded at: https://www.mdpi.com/article/10.3390/cancers14112807/s1, Figure S1: SCAMP3 regulates gene expression of EGFR and PDGF pathways.; Figure S2: Full Western blot images. Table S1: In vitro effects of depletion of SCAMP3 on the expression of EGF/PDGF signaling pathway genes.
