# Supplementary material for: SCAMP3 Regulates EGFR and Promotes Proliferation and Migration of Triple-Negative Breast Cancer Cells through the Modulation of AKT, ERK, and STAT3 Signaling Pathways
Source: Cancers (Basel). 2022 Jun 5;14(11):2807. doi: 10.3390/cancers14112807 (PMC9179572; doi:10.3390/cancers14112807)
Supplement: Supplementary file 1 [file cancers-14-02807-s001.zip › Supplemental Figure S1.pdf]

(A)

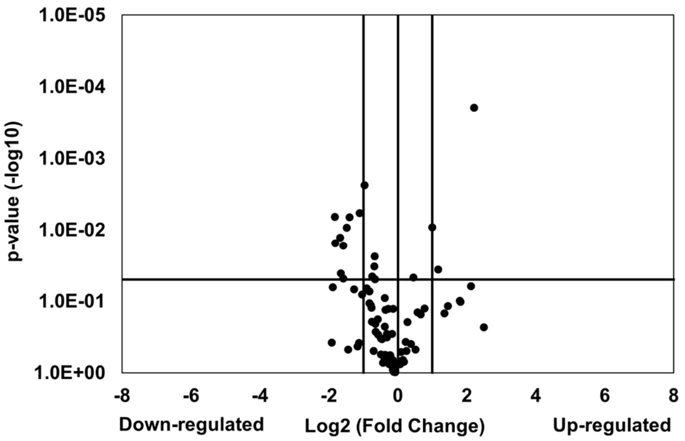

(B)

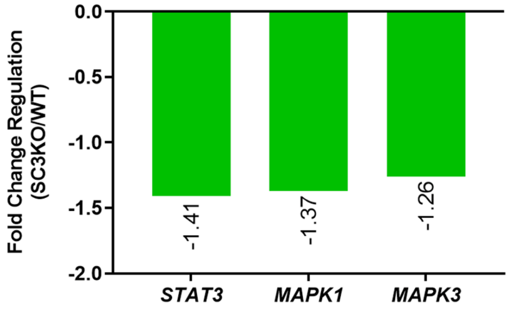

Figure S1. SCAMP3 regulates gene expression of EGFR and PDGF pathways. RT² PCR arrays to profile the expression of EGF/ PDGF Signaling Pathway specific genes were performed to three different samples from SUM-149 WT and SC3KO cells. ACTB, B2M, GAPDH, HPRT1 and RPLP0 were used as reference genes. (**A**) Volcano plot shows the effects on gene expression analyzed at -2.0 ≥ 2.0 log2-fold change (vertical lines). Statistically significant ($p < 0.05$) regulated genes are above the horizontal black line. (**B**) Gene expression of *STAT3*, ($p = 0.06$), *MAPK1* ($p = 0.08$) and *MAPK3* ($p = 0.03$).
