# Supplementary material for: SCAMP3 Regulates EGFR and Promotes Proliferation and Migration of Triple-Negative Breast Cancer Cells through the Modulation of AKT, ERK, and STAT3 Signaling Pathways
Source: Cancers (Basel). 2022 Jun 5;14(11):2807. doi: 10.3390/cancers14112807 (PMC9179572; doi:10.3390/cancers14112807)

Figure S2. Full Western blot images.
Western Blotting in Bands in Figure 1

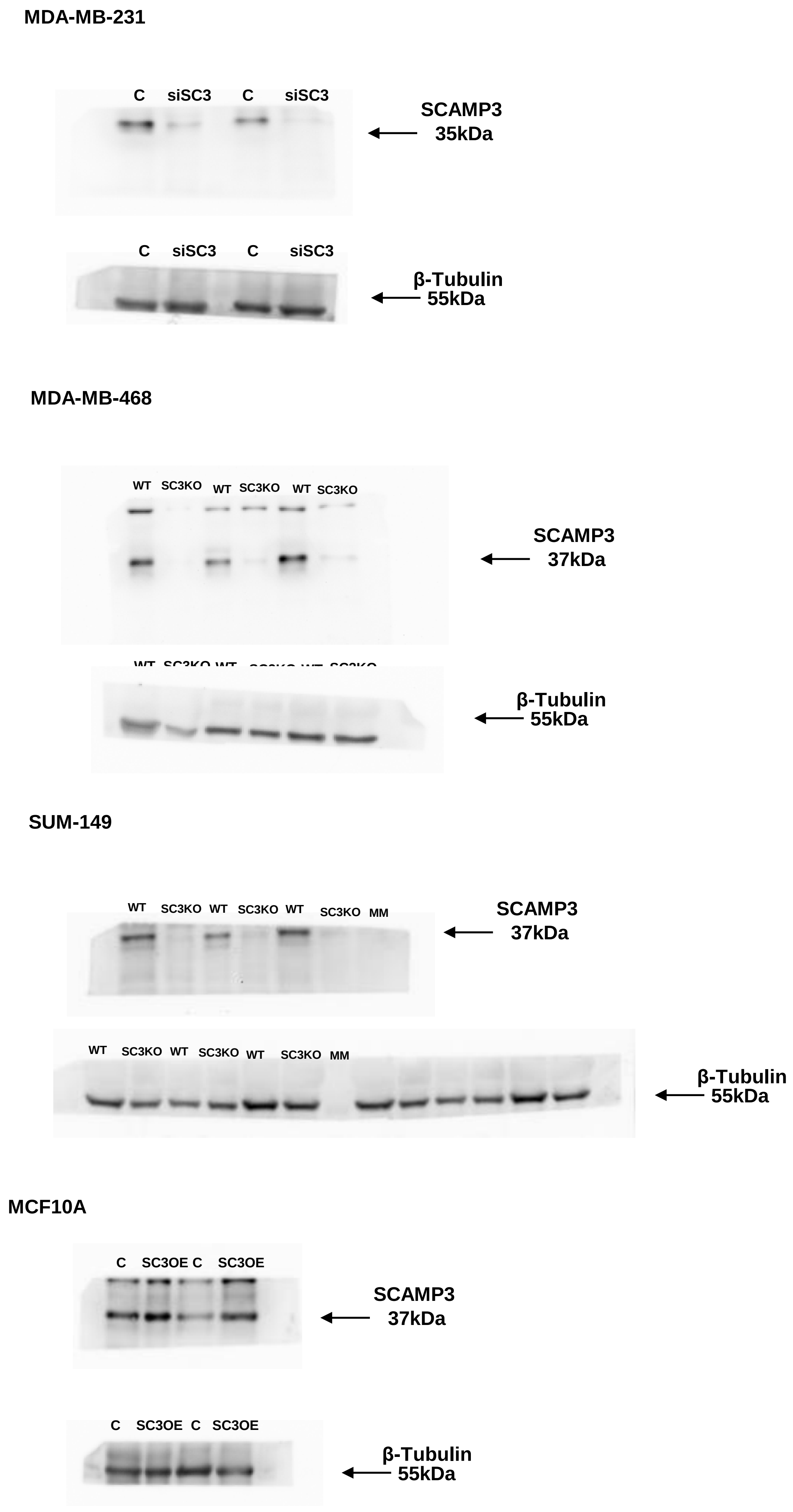

Western Blotting in Bands in Figure 2A

MDA-MB-231

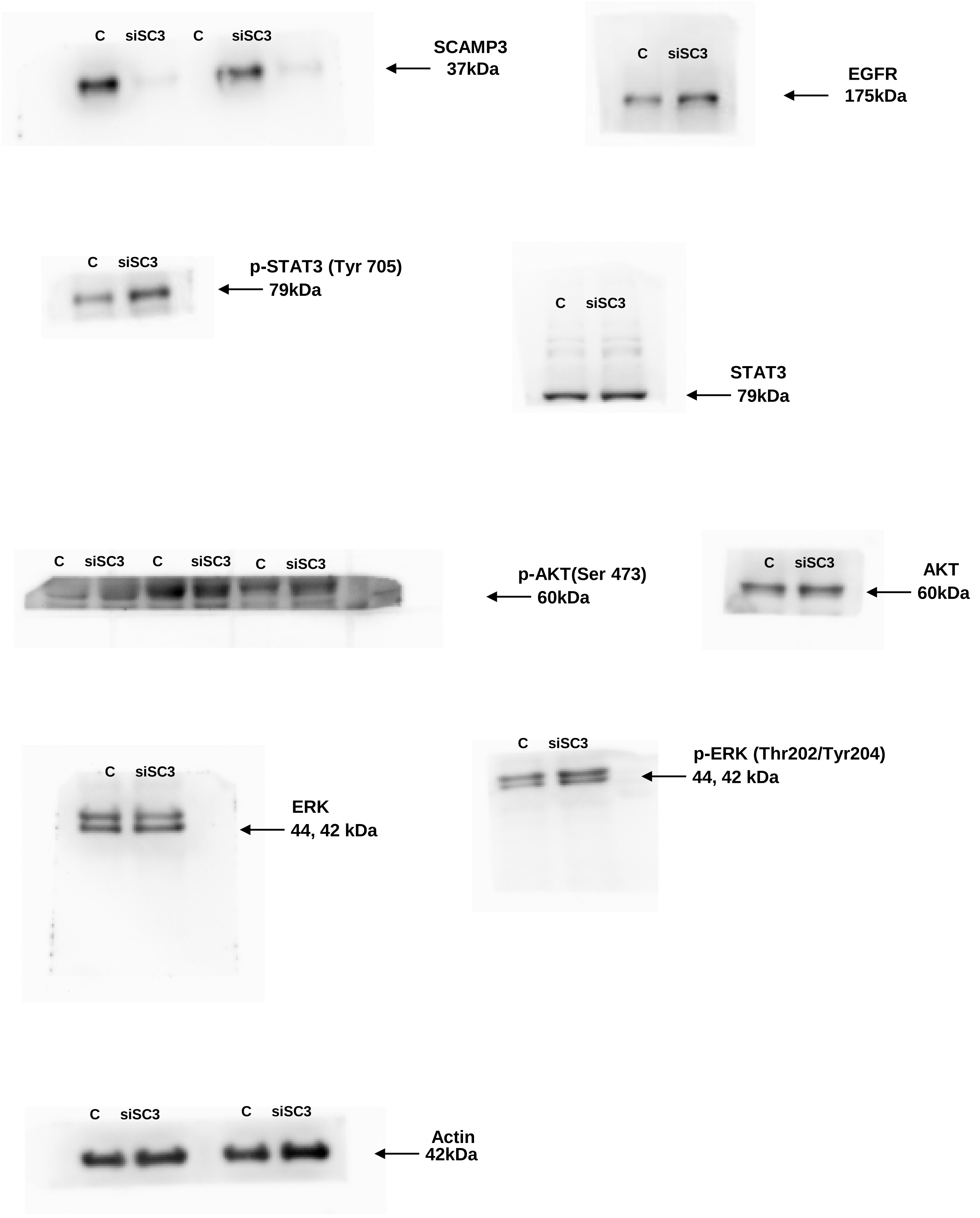

Western Blotting in Bands in Figure 2B

MDA-MB-468

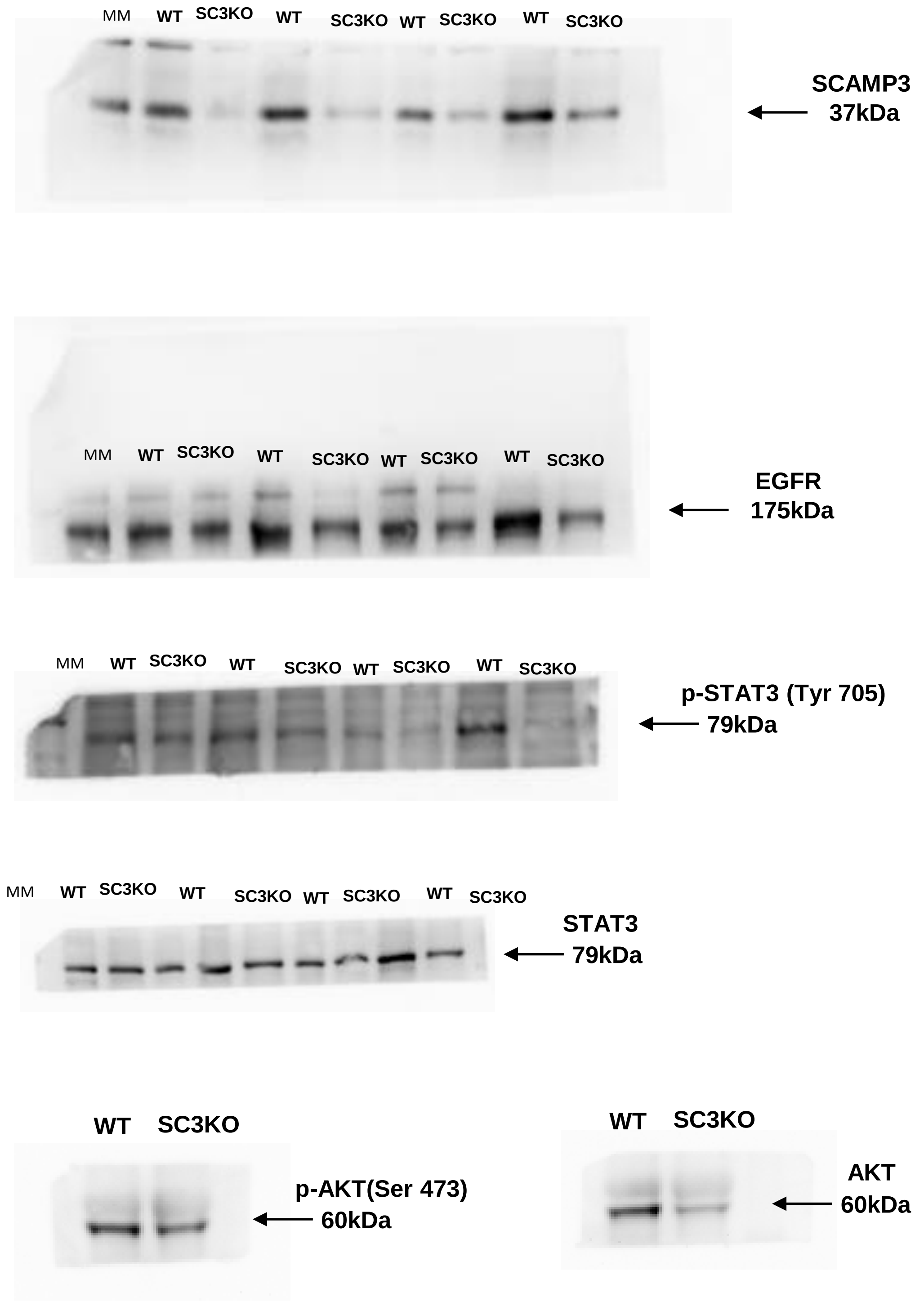

Western Blotting in Bands in Figure 2B

MDA-MB-468

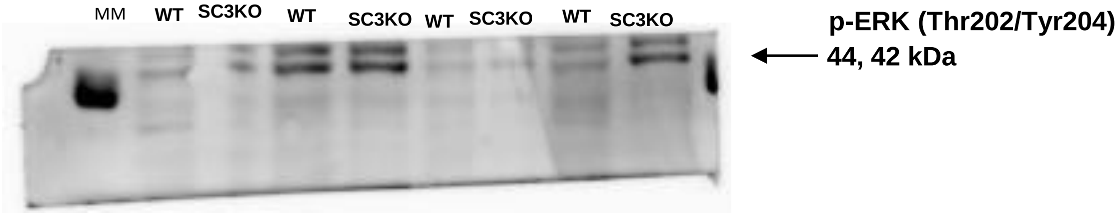

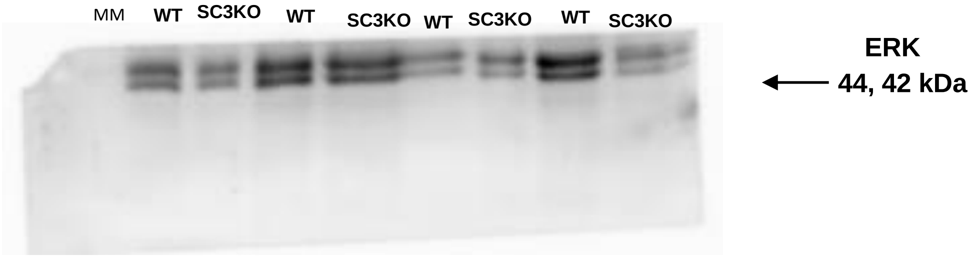

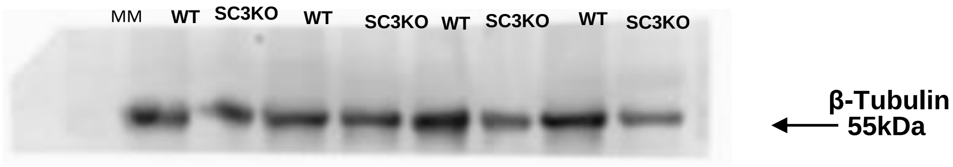

Western Blotting in Bands in Figure 2C

MCF10-A

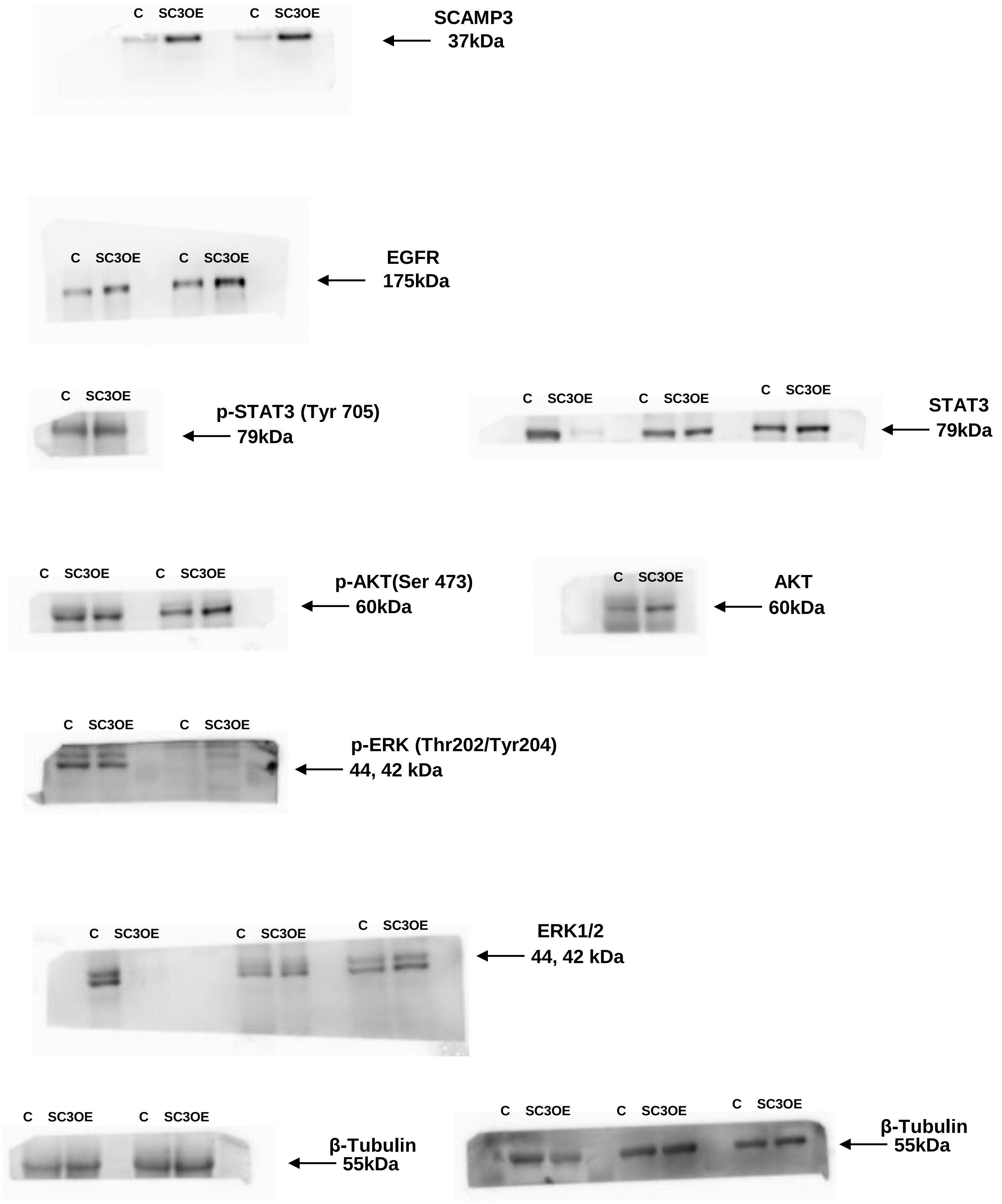

Western Blotting in Bands in Figure 5

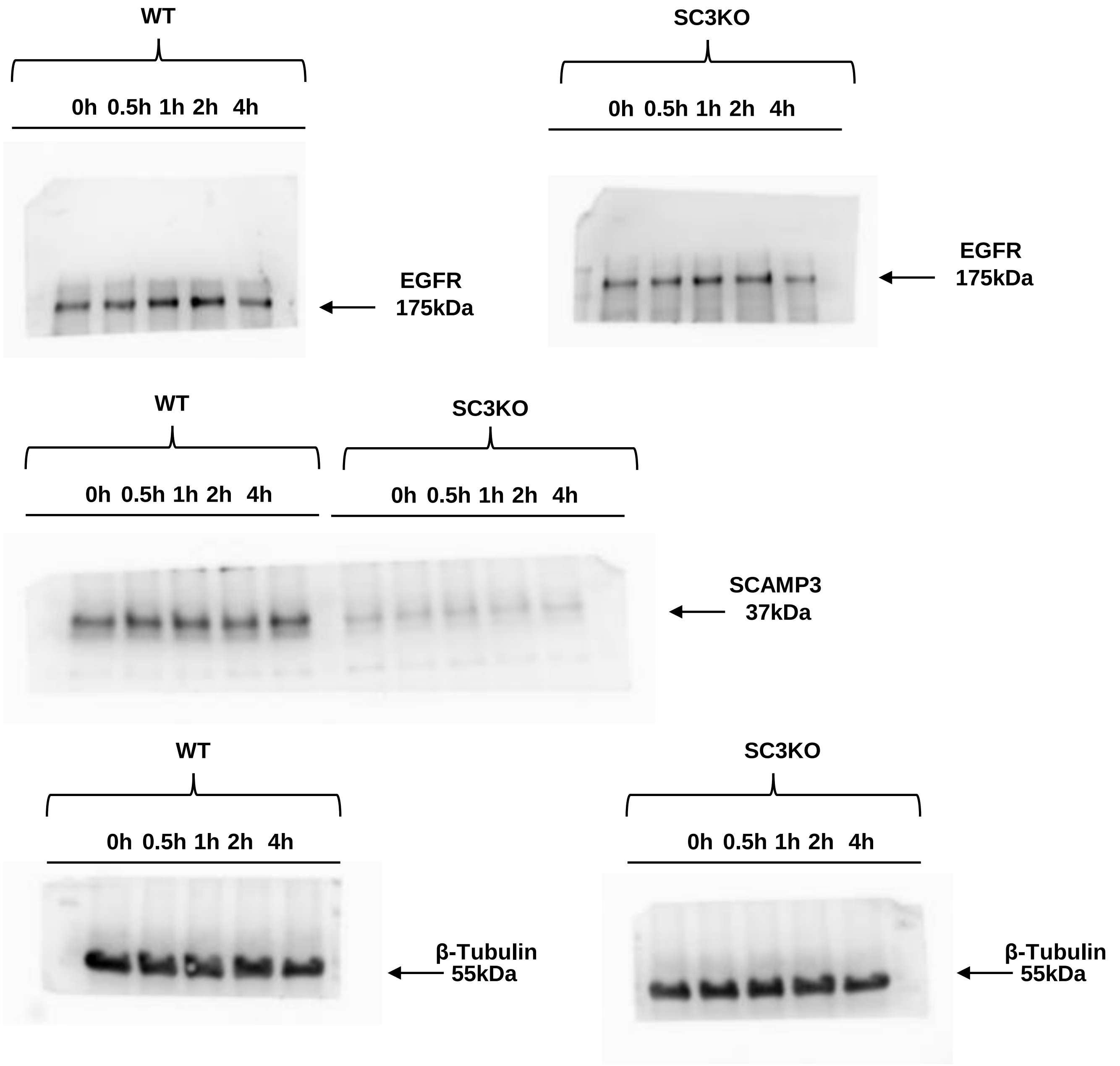

Western Blotting in Bands in Figure 5

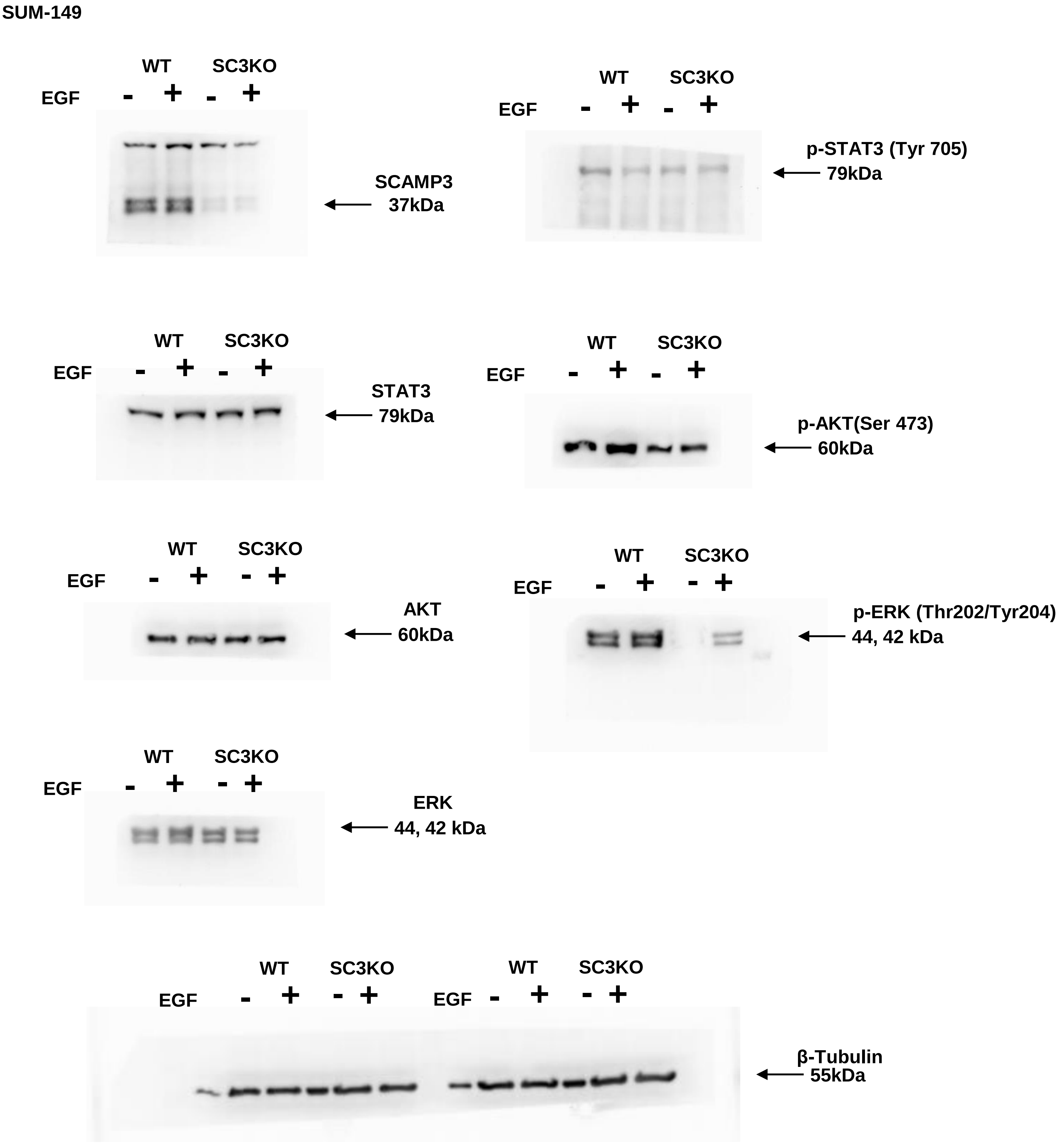

Western Blotting in Bands in Figure 6

Tumors

WT

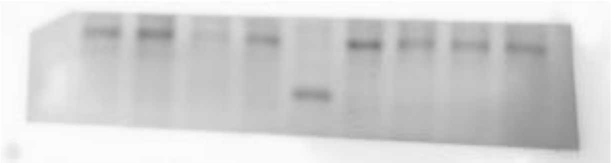

SCAMP3
35kDa

SC3KO

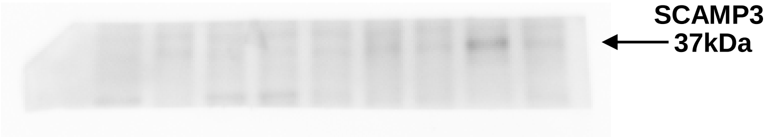

SCAMP3
37kDa

WT

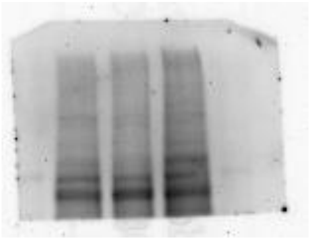

pEGFR
175kDa

SC3KO

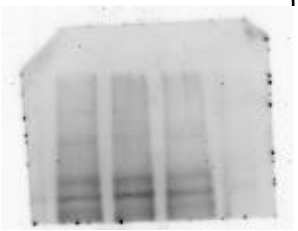

pEGFR
175kDa

WT

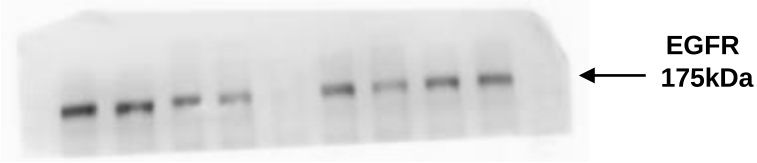

EGFR
175kDa

SC3KO

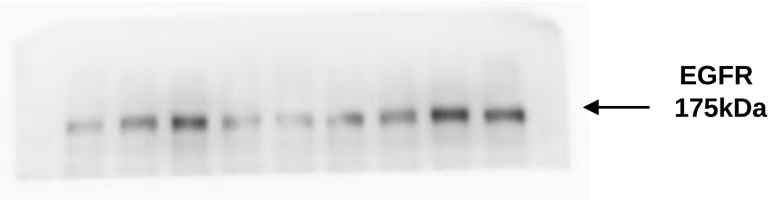

EGFR
175kDa

WT

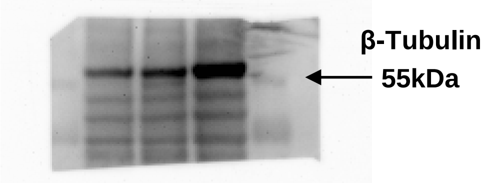

β-Tubulin
55kDa

SC3KO

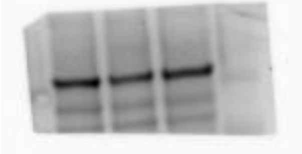

β-Tubulin
55kDa

Western Blotting in Bands in Figure 6

Tumors

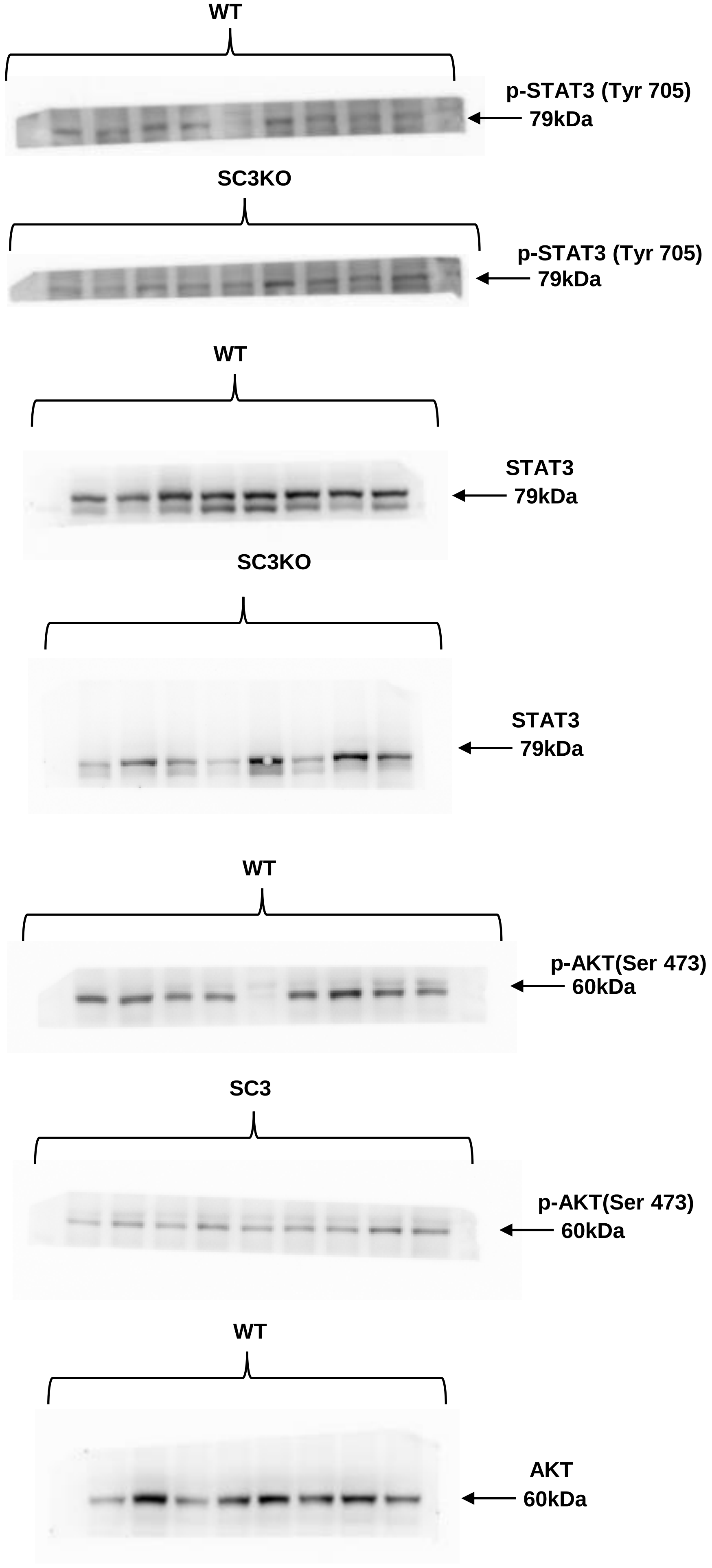

Western Blotting in Bands in Figure 6

Tumors

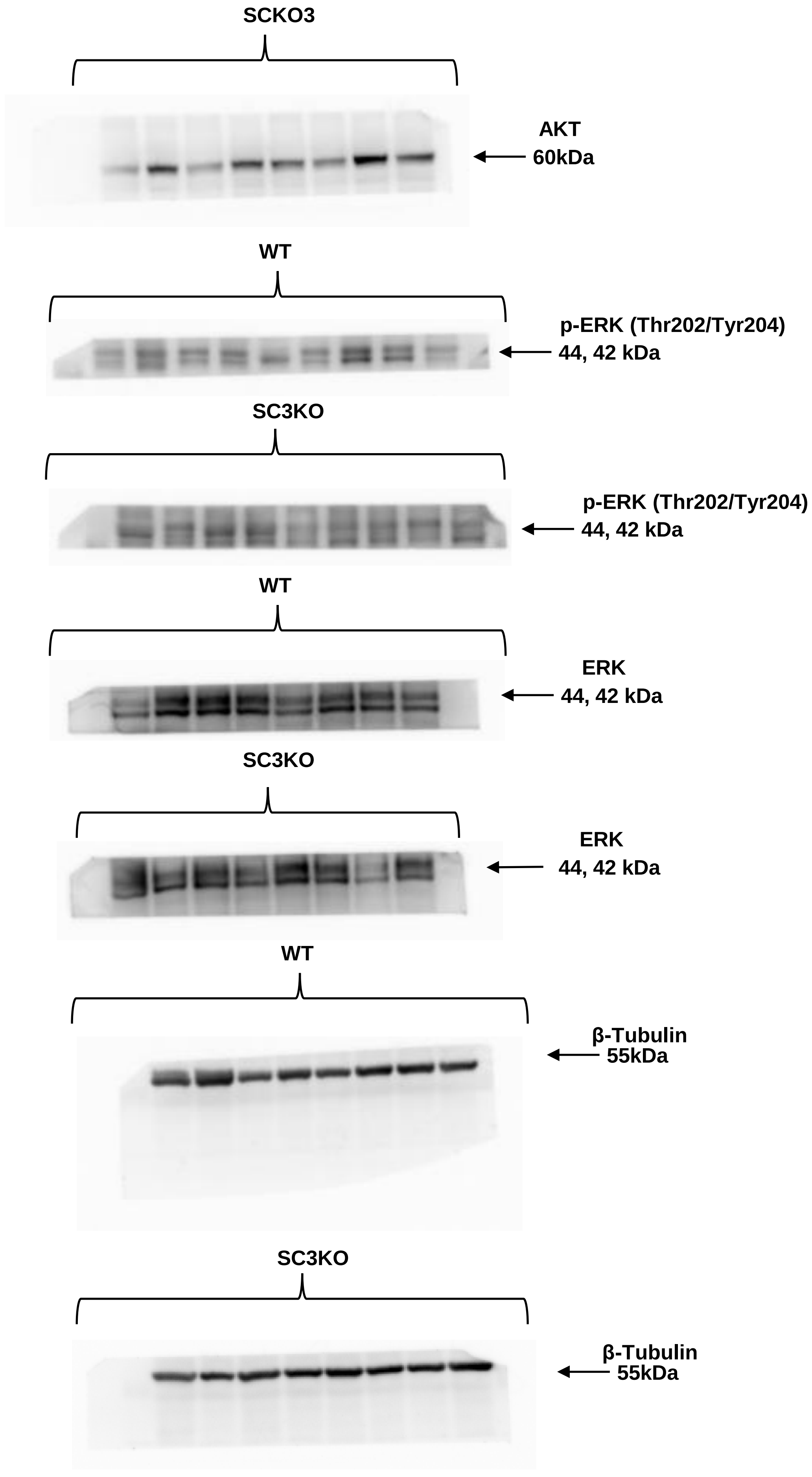

Supplement: Supplementary file 1 [file cancers-14-02807-s001.zip › Supplemental Figure S2.pdf]
