# Supplementary material for: SCAMP3 Regulates EGFR and Promotes Proliferation and Migration of Triple-Negative Breast Cancer Cells through the Modulation of AKT, ERK, and STAT3 Signaling Pathways
Source: Cancers (Basel). 2022 Jun 5;14(11):2807. doi: 10.3390/cancers14112807 (PMC9179572; doi:10.3390/cancers14112807)
Supplement: Supplementary file 1 [file cancers-14-02807-s001.zip › Supplemental Table S1.pdf]

Table S1. *In vitro* effects of depletion of SCAMP3 on the expression of EGF/PDGF signaling pathway genes.

**Symbol**	**Name**	**Fold Change***	***p*-value***
AKT2	V-akt murine thymoma viral oncogene homolog 2	-3.6	0.006
BCAR1	Breast cancer anti-estrogen resistance 1	-1.6	0.02
BCL2	B-cell CLL/lymphoma 2	2.0	0.009
BRAF	V-raf murine sarcoma viral oncogene homolog B1	-2.0	0.002
CASP3	Caspase 3, apoptosis-related cysteine peptidase	-2.8	0.009
DUSP6	Dual specificity phosphatase 6	-1.6	0.05
EGR1	Early growth response 1	2.2	0.04
EPS8	Epidermal growth factor receptor pathway substrate 8	-2.6	0.007
GSK3A	Glycogen synthase kinase 3 alpha	-3.2	0.01
MAP2K1	Mitogen-activated protein kinase kinase 1	-2.2	0.006
MAPK10	Mitogen-activated protein kinase 10	4.6	0.0002
PDGFB	Platelet-derived growth factor beta polypeptide	-3.2	0.04
PRKCA	Protein kinase C, alpha	-1.6	0.03
RASA1	RAS p21 protein activator (GTPase activating protein) 1	-1.7	0.04
SHC1	SHC (Src homology 2 domain containing) transforming protein 1	-3.0	0.05
STAT5A	Signal transducer and activator of transcription 5A	-3.6	0.02

* Table shows genes up- and down-regulated ≥1.5-fold change and $p \leq 0.05$.
